# Gender preference gaps and voting for redistribution

**DOI:** 10.1007/s10683-021-09741-8

**Published:** 2022-01-06

**Authors:** Eva Ranehill, Roberto A. Weber

**Affiliations:** 1 Department of Economics, University of Gothenburg, Gothenburg, Sweden; 2 Department of Economics, Lund University, Lund, Sweden; 3 Department of Economics, University of Zurich, Zurich, Switzerland

**Keywords:** Gender differences, Risk, Altruism, Redistributive preferences, Experiment, C91, C92, J16, H23

## Abstract

There is substantial evidence that women tend to support different policies and political candidates than men. Many studies also document gender differences in a variety of important preference dimensions, such as risk-taking, competition and pro-sociality. However, the degree to which differential voting by men and women is related to these gaps in more basic preferences requires an improved understanding. We conduct an experiment in which individuals in small laboratory “societies” repeatedly vote for redistribution policies and engage in production. We find that women vote for more egalitarian redistribution and that this difference persists with experience and in environments with varying degrees of risk. This gender voting gap is accounted for partly by both gender gaps in preferences and by expectations regarding economic circumstances. However, including both these controls in a regression analysis indicates that the latter is the primary driving force. We also observe policy differences between male- and female-controlled groups, though these are substantially smaller than the mean individual differences—a natural consequence of the aggregation of individual preferences into collective outcomes.

**Supplementary Information:**

The online version contains supplementary material available at 10.1007/s10683-021-09741-8.

## Introduction

It is often claimed that the world, if run by women, would be very different than it is today (Funk & Gathmann, 2015).^1^ However, since men have traditionally dominated cultural, political and economic decision making in most societies, we know very little about which institutions, policies and social outcomes would result if women were in charge. Nevertheless, as women become more involved and influential in corporate and policy decision making, it is important to better understand whether they do, in fact, implement distinct policies than those implemented by men.

The notion that having women in control of policymaking would produce a fundamentally different world is often supported by the observation, in the academic literature, of gender gaps in important areas of economic decision making and psychological traits (Bertrand, 2011; Croson & Gneezy, 2009; Niederle, 2016). This literature finds women to be, on average, less risk taking, less confident and less willing to compete than men (e.g., Niederle & Vesterlund, 2007; Eckel and Grossman, 2008; Niederle & Vesterlund, 2010; Falk and Hermle, 2018). In the context of pro-sociality, women are often relatively more concerned with equality than efficiency relative to men (e.g., Almås et al., 2010; Andreoni & Vesterlund, 2001; Sutter et al., 2010). It seems plausible that preferences for less risk, competition and inequality would lead female-dominated societies to also be more equitable, more secure and less competitive. Indeed, other studies have documented that women tend to favor more progressive redistributive policies (Alesina and Giuliano, 2011; Funk & Gathmann, 2015), which may be a manifestation of such gender gaps in more basic preferences.

However, while the observation of gender gaps in basic preferences presents a plausible channel through which greater involvement in policymaking by women may yield different policies and outcomes, more evidence about the precise relationships between such basic preferences and policy related behaviors, such as voting, are needed. For example, it is necessary to document that differences in the voting behavior of men and women are truly the result of stable preference differences for things like equality and security, rather than of economic circumstances that may lead women to benefit from more redistribution and a larger social safety net. In the latter case, gender gaps in policy preferences may diminish as women gain improved economic and political standing. Moreover, even if there is a channel from basic preferences to voting, it is also necessary that such influence be sufficiently strong and robust to be consistently manifested in substantially different policy outcomes as the share of women holding decision-making authority increases.

Indeed, while several studies find that having women in power produces different policies and outcomes (e.g., Chattopadhyay & Duflo, 2004; Matsa & Miller, 2013), other studies fail to find such differences (e.g., Adams, 2016; Eckbo, Nygaard and Thorburn, 2016; Campa and Bagues, 2021). The absence of stronger relationships may be because women do not inherently favor different kinds of policies or outcomes based on deep-seated underlying preference differences, or because decision-making institutions mitigate any differences in the manifestation of such preferences even as the share of female decision-makers increases.

In this paper, we use the control provided by laboratory experiments to investigate gender gaps in policy preferences and to understand better what factors are related to such gaps.^2^ In order to study voting behavior by gender in a manner that captures the basic features of many real-world policy decisions, we design a context in which groups of participants engage in real-effort production and endogenously determined redistribution. In each period of the main part of our experiment, group members first vote for their preferred redistributive policy—analogous to a linear tax rate—before engaging in production. Thus, participants state their preferred redistribution rule before they know their realized task income. In this setting, by means of their vote, participants can support egalitarian redistributive policies that decrease competition and risk and create greater equality or regressive policies that do the opposite. Our design thus yields a straightforward directional hypothesis for how previously documented gender gaps in preferences for risk, competition and equality should translate into policy preferences: women will vote for more egalitarian redistributive policies than men.

To better understand the basis for potential gender voting gaps, we also investigate the extent to which such gaps interact with the riskiness of the production environment. Our design varies by treatment whether individuals’ output translates into wealth deterministically, meaning there is no risk, or is subject to random shocks that add risk to the relationship between productivity and income. This allows us to investigate whether women—who prior evidence suggests tend to be more risk averse—exhibit a greater preference for more egalitarian policies, relative to men, as the riskiness of the production environment increases.

Consistent with the hypothesis that gender preference gaps translate into policy preferences we observe large gaps in voting behavior, with women preferring substantially more egalitarian redistributive policies than men. This gap persists over time, as participants gain experience and feedback in the production and redistribution environment. Moreover, while we find that participants vote for more egalitarian redistribution policies when the environment is inherently riskier, the gender gap in voting remains very similar both under low and high risk. Thus, differential risk preferences between men and women do not seem to result in varying gender gaps in demand for redistribution under varying degrees of risk, suggesting that gender gaps in risk preferences may not be a fundamental driver of the gender voting gap.

We then investigate more directly the degree to which gender gaps in voting are related to various individual preferences for which earlier research has documented gender gaps. We employ incentivized and unincentivized measures of risk preferences, social preferences and competitiveness. In each case, we confirm the typical behavioral gender gaps in the literature—women are more risk averse, less willing to compete and prioritize equality relative to efficiency to a greater degree than men. We then use these measures to test the extent to which these individual preference gaps account for the gender gap in voting for redistributive policies. We find that introducing these preferences into our regressions reduces part of the voting gap—roughly 35 percent of the total gender gap in the first period and 22 percent of the gap in subsequent periods.

We also measure individual performance on the production task at the beginning of the experiment, before beginning the group activity involving voting and redistribution, and beliefs about relative performance. Consistent with earlier evidence that women are less overconfident than men, we find that women have less positive expectations regarding their relative performance on this initial task, even though actual performance does not differ by gender. Importantly, measures of relative performance beliefs account for between 40 and 50 percent of the gender gap in voting, suggesting that a large part of the gender gap is due to differential expectations of performance—and, therefore, income—in the production task. Moreover, when including controls for both preferences and performance beliefs in our regression analysis, gender preference gaps have only a limited impact on the gender voting gap, suggesting that differential expectations regarding economic circumstances are the primary driving force behind the gender voting gap.

Finally, we study the extent to which the resulting group policies—which are determined in each period by the median vote in a group—vary depending on whether men or women are in majority.^3^ While it is natural to assume that a difference in the average preferences for a particular policy between men and women will lead policy outcomes in female- versus male-majority groups to reflect such differences, it is straightforward to show that collective decision making and intra-gender heterogeneity may often dampen these effects.^4^ Our results confirm this mechanical attenuation of the gender difference in policy preferences. The impact on policy outcomes of a group having a male or female majority is substantially smaller—by about half—relative to the difference between the individual preferences of men and women. Moreover, over the course of the 10 periods of our experiment, the gap in policies implemented in male- versus female-majority groups is not reliably statistically significant. While our experiment is only illustrative in this regard—since there could be many alternative social choice mechanisms for setting redistributive policy—it provides a demonstration that the gaps produced in female- versus male-majority decision-making bodies are likely to be smaller than the underling gender preference gaps.

Our results demonstrate that while part of the persistent and substantial gender gap in voting for redistribution can be connected to underling gender preference gaps—primarily for less competition and more equality—the gender gap in relative performance beliefs is the most important underlying factor. Our work thus indicates that gender gaps in preferences may have some influence on behavior and policy outcomes as women’s participation in policymaking grows. However, our findings also suggest that this impact is secondary to that of beliefs about relative economic outcomes, which may change as women attain greater economic equality.

Moreover, the gaps in policy outcomes between male-majority and female-majority groups are substantially smaller than the gaps in male versus female policy preferences. Thus, we also provide interpretations for why many studies fail to find different outcomes as the gender composition of decision-making bodies changes: women may be less fundamentally motivated to pursue different policies than one might think by looking at their voting behavior, and the attenuating properties of collective decision-making rules may further dampen the effects of varying gender composition.

The remainder of this article is structured as follows. Section 2 discusses related literature. Section 3 describes the experiment design. Section 4 presents the results, first focusing on gender gaps in voting, then on the extent to which more basic gender preference gaps can explain the voting gap and, finally, on whether group outcomes differ between male-majority and female-majority groups.^5^ Section 5 concludes.

## Related literature

Our study relates to several different strands of research. One of these strands explores the extent to which women vote for different policies than men. While some studies indicate that women favor different policies than men, there is no consensus whether differences arise because women are intrinsically different than men, or whether they face different economic circumstances. For example, Funk and Gathmann (2015) and Alesina and Giuliano (2011) find that women in Switzerland and the US tend to have more favorable attitudes toward redistribution and to prioritize policies such as welfare more than men. This result holds even after controlling for a range of socio-economic characteristics, suggesting something intrinsically female about such policy preferences. However, Edlund and Pande (2002) find that the emergence of a gender gap in political preferences in the US from 1983 to 2003—with women voting more for left-leaning policies—strongly correlates with the decline in marriage. This leads the authors to speculate that the gender gap in political preferences results from higher divorce rates making men wealthier and women poorer.

Another related body of literature explores to what extent female leaders make different decisions than men, also finding somewhat contradictory results. For example, Chattopadhyay and Duflo (2004) study random political reservations for women in India and find that the public goods provided in villages with a female council head are more sensitive to the priorities of female constituencies. However, Campa and Bagues (2021) find no impact of gender quotas in candidate lists in local Spanish elections on the size or composition of public spending. Exploring the impact of female representation in the private sector, Matsa and Miller (2013) find that companies affected by a Norwegian quota requiring a minimum of 40% female board members experience higher labor costs—due to fewer layoffs compared to companies unaffected by the quota—and lower operating profits. However, Eckbo et al. (2016) question Matsa and Miller’s findings (2013) by arguing that extending the sample period generates a non-significant effect of the quota on company value.^6^ When taken together, evidence of an impact of increased representation of female decision-makers is thus mixed. Moreover, some of this research suggests that, rather than fundamentally different societies—less competitive, less risky, more egalitarian—female policy control may mainly produce societies that prioritize policies more directly beneficial to women.^7^

Implicitly, this literature connects the impact of a larger share of female decision-makers on outcomes with gender preference gaps, although there is no clear measurement of these policymakers’ or managers’ preferences or beliefs, nor of the extent to which these traits are responsible for differences in enacted policies. It is, however, an important connection to test, given that it is relevant for understanding the nature and stability of gender gaps in preferences for specific policies and outcomes.

A large body of research documents gender gaps in economic preferences related to risk, competitiveness and pro-sociality, as well as relative performance beliefs (for reviews, see Croson & Gneezy, 2009; Bertrand, 2011; Niederle, 2016). While many studies find support for systematic gender gaps (e.g., Pulford and Colman 1997; Niederle & Vesterlund, 2007; Eckel and Grossman, 2008; Falk et al., 2018), some recent literature raises questions about their generality and magnitude. For instance, recent reviews argue that the gender gap in risk attitudes may be smaller and less reliable than previously thought (Filippin & Crosetto, 2016; Niederle, 2016; Nelson, 2015). Similarly, while many studies find a large gender gap in the willingness to enter competitive environments, most of these studies rely on a common, math-based, paradigm inspired by Niederle and Vesterlund’s (2007) seminal article. Other studies indicate that gender differences in competitiveness sometimes disappear—for example, in tasks that are not male stereotyped (Cárdenas et al., 2012; Dreber et al., 2014; Günther et al., 2010; Grosse, Riener, and Dertwinkel-Kalt 2014; Shurchkov, 2012, although see also, e.g., Wozniak et al., 2014), when time pressure is reduced (Shurchkov, 2012), or when information about relative performance is available (Ertac and Szentes, 2011; Wozniak et al., 2014; though Cason et al., 2010 find the opposite result). Turning to pro-sociality, meta-analyses of dictator-game giving by Engel (2011) and Bilén, Dreber and Johannesson (2020) find only small gender differences. However, several studies suggest that men and women differ in their preferences for efficiency versus equality, rather than in general pro-sociality (Almås et al., 2010; Andreoni & Vesterlund, 2001; Sutter et al., 2010). Finally, a large literature indicates that women are often less confident in their ability than are men, but this finding is sometimes moderated by the stereotype associated with the relevant task (e.g., Lundeberg et al., 1994; Barber and Odean, 2001; Niederle & Vesterlund, 2007).^8^

Perhaps most closely related to our work are a few studies that explore the relationships between basic preferences for risk and equality and political preferences, finding some evidence that such basic preferences may partly explain gender gaps in policy preferences. Gärtner et al. (2017) survey a representative sample of 1,365 Swedish adults to study the relationship between risk preferences and general support for redistribution. They measure risk preferences using eight hypothetical choices between a safe amount and a lottery and measure attitudes toward redistribution by asking, “How much economic redistribution do you want in society?” Controlling for other observable characteristics, women exhibit slightly more support for redistribution, but this relationship is not robustly statistically significant. Introducing the risk aversion measure—which significantly predicts attitudes toward redistribution—decreases the magnitude of the gender coefficients by about 15 percent.^9^

Fisman et al. (2017) use a web survey to elicit the distributional preferences of a large sample of Americans. They then explore the extent to which the resulting preference types exhibit differential support for Barack Obama and the Democratic Party in the 2012 Presidential Election. While their study is not explicitly about gender, women in their sample are more likely to prioritize equality over efficiency, though the statistical significance of this relationship is not robust to corrections for multiple hypothesis testing. Women are also more likely to report having voted for Obama and for the Democratic party—though neither relationship is statistically significant. Controlling for distributional preferences decreases the relationships between gender and voting for Obama and Democrats by 14 percent and 19 percent, respectively.^10^

Hence, these studies find some evidence of gender differences in preferences for risk and for equality versus efficiency and, in exploratory analysis, identify that these preference gaps may have an impact on support for statements favoring redistribution or for a specific left-leaning political candidate. But they do not clearly demonstrate that women vote for different policies than men, nor are they designed to investigate, more broadly, the degree to which gender gaps in preferences and expectations influence policy preferences.

Finally, only a few studies in economics investigate the extent to which gender differences in policy preferences at the individual level persist through collective decision-making.^11^ Dufwenberg and Muren (2006) explore the relationship between group gender composition and sharing decisions in the dictator game, finding that female-majority groups are more generous than male-majority groups. A few studies explore whether the existence and size of speculative bubbles in experimental asset markets depend on the gender composition of the traders. Eckel and Füllbrunn (2015) find an inverse relationship between the magnitude of price bubbles and the share of female traders. However, Cueva and Rustichini (2015) find all measures of mispricing to be comparable, or worse, in all-female markets than in all-male markets, while Eckel and Füllbrunn (2017) find no differences when subjects are not informed of the market gender composition. Moreover, Eckel and Füllbrunn (2015) find that gender gaps in individual-level preferences, such as risk aversion, have weak and statistically insignificant relationships with bubble formation. Hence, these studies provide only modest evidence of links between basic behavioral gender gaps and the outcomes produced by male- versus female-controlled groups.

## Experimental design

Our experiment consisted of three parts.^12^ In Part 1 we elicited individual preferences related to risk and concern for others. Participants also answered questions about their age and gender. In Part 2 we elicited participants’ baseline productivity in the production task, as well as their preferences for competition and their relative performance beliefs. Participants received no information about outcomes or earnings for Parts 1 and 2 until the end of the study.

In the main part, Part 3, participants performed the production task with redistribution in fixed groups of five, for 10 periods. Groups varied with respect to their gender composition, although at no point were participants made aware of the identity or gender of their group members.^13^ Finally, after Part 3, we once again elicited individual productivity. Subjects also completed an exit questionnaire.

The experiment comprised two conditions, which varied only with respect to how participants earnings were generated. Specifically, the conditions varied whether participants were paid a fixed piece rate for their output (the *No Risk* condition), or whether individuals’ income was subject to random shocks (the *Risk* condition). We first describe the experiment as implemented in the No Risk condition, and then explain how the Risk condition differs.

### Parts 1 and 2: Preferences, productivity, and performance beliefs

We began by eliciting a variety of individual preferences using both incentivized and non-incentivized measures. Participants did not receive feedback about the outcome in any of the measures elicited in Parts 1 and 2 until the end of the study. We elicited these preference measures before the main part of the study (Part 3), since this subsequent part provided full feedback about relative performance and economic outcomes, which would likely have contaminated the basic preference measures.

As an incentivized measure of risk preferences we implemented the investment game of Gneezy and Potters (1997) in which participants allocated a portion of an initial endowment of 100 Experimental Currency Units (ECU) to a risky investment. The investment failed with a probability of 50%, in which case the invested money was lost, while with 50% probability the investment returned 2.5 times the invested amount. We also elicited risk preferences through a non-incentivized survey question about general risk-taking propensity introduced by Dohmen et al. (2011).

We also elicited social preferences using two incentivized measures and one non-incentivized survey question. For the incentivized measures we used the full version of the Social Value Orientation scale (Murphy et al., 2011), in which participants make 15 choices allocating wealth between one’s self and another randomly selected participant. Choices in the first 6 decisions (the “Primary” dimension) allow a classification of a participant’s type along a spectrum of pro-social motivations, from competitive to altruistic. The remaining 9 decisions (“Secondary”) identify a subject’s willingness to trade off equality versus efficiency. As a non-incentivized measure, we administered a hypothetical question about how much a participant would donate to charity if he or she unexpectedly received 1000 Swiss Francs (Dohmen et al., 2011).

**Fig. 1:**
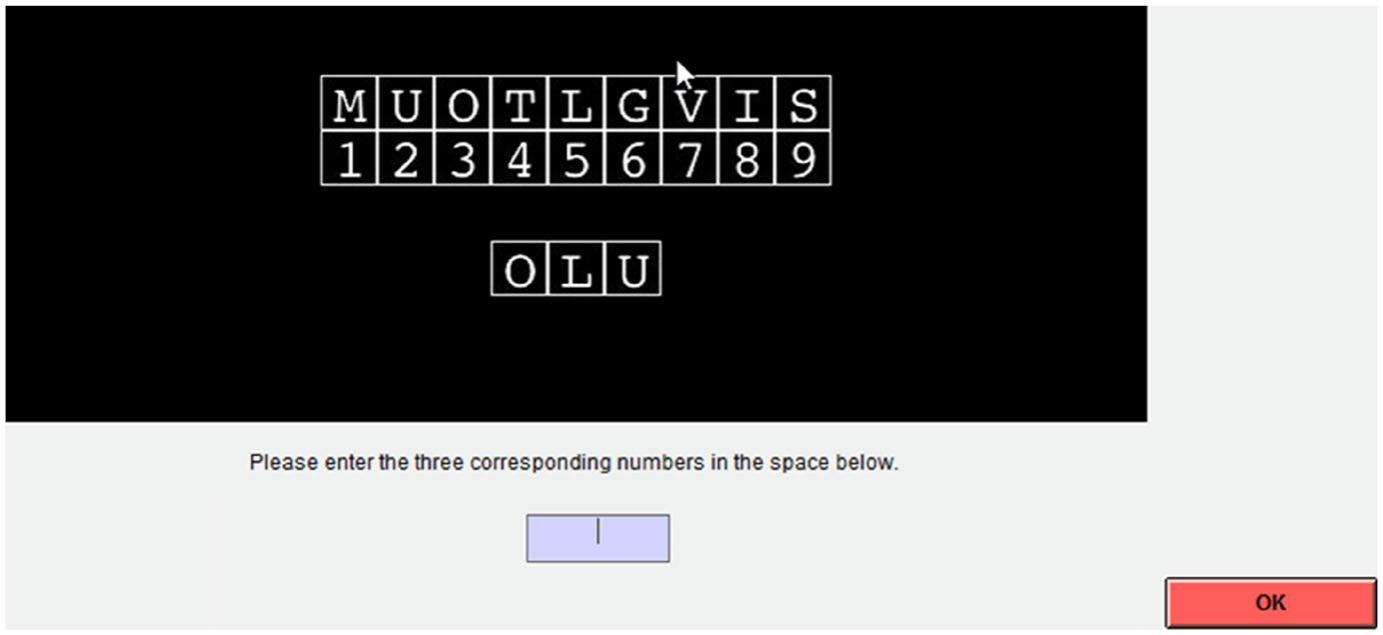
The digit-letter substitution task

In Part 2, participants performed the real-effort production task that would form the basis of the main part of the experiment once under piece-rate incentives to provide us with a measure of individual productivity. The task was a computerized version of a digit-substitution task (Iriberri and Rey-Biel, 2011; Erkal et al., 2011).^14^ Participants were shown keys, consisting of a unique mapping of 9 letters to numbers, and could decode sequences of three letters into numbers (see Fig. 1). Keys were changed every ninth three-letter sequence. If a sequence was decoded incorrectly, a participant had to decode the same sequence until the entry was correct. Participants had 90 s to decode as many sequences as possible and received a payment of 10 ECU for each correct entry.

After participants performed the task under piece-rate incentives, we elicited their willingness to engage in competition. To save time, the measure implemented differs from the measure introduced by Niederle and Vesterlund (2007) in that participants were asked to choose between piece-rate or tournament pay for their previous performance instead of performing the task again. Under the piece rate, a participant received a second payment identical to his or her earlier payment for performance (10 ECU per correctly completed entry). Alternatively, under the competitive payment, the participant’s score was compared to that of a randomly selected other participant and yielded either double the original piece-rate payment (20 ECU per correct entry) if the participant’s performance was higher or, otherwise, yielded nothing. Ties were broken randomly. This binary choice is our measure of competitiveness.

We also elicited subjects’ beliefs about their relative task performance. At the conclusion of Part 2, participants guessed their performance rank in the task relative to other participants in the experimental session. Accurate responses were incentivized: participants received an extra payment of 50 ECU if their guessed rank was within two of their actual rank.^15^

### Part 3: Repeated production with redistribution

At the beginning of Part 3, participants were randomly assigned to five-person groups. These groups then remained the same for the 10 periods of Part 3.

In each period, groups performed the same activity involving voting, production and redistribution. Participants first voted for their preferred redistribution policy and then observed the policy implemented for their group in that period. Group members thereafter engaged in the real-effort production task and generated income according to the piece-rate scheme as in Part 2. Once the production phase was over, this income was subject to redistribution per the policy determined at the beginning of the period. At the end of the period, participants received detailed information about individual outcomes for all group members, as described below.

#### The vote

At the beginning of each period, all five group members simultaneously cast a vote for a redistribution parameter, $t\in [-1.00, 1.00]$, analogous to a linear tax rate. Following the vote, all group members observed the resulting redistribution parameter, determined by the median vote, which would be applied to the group earnings at the end of the relevant period. Using the median vote, in contrast to using, for example, the average vote, implies that each participant is incentivized to provide his or her preferred value of $t$, reducing strategic voting. This fact was also stressed in the instructions.

#### Production

After the vote, participants worked independently on the real-effort production task. As in Part 2, each production period lasted 90 s, and each correctly completed entry generated 10 ECU of income. In the Risk condition, this certain 10 ECU was replaced by a stochastic payment, as described below.

#### Redistribution

Following production in a period, task income was redistributed according to the implemented redistribution policy for that period. Given a policy, $t$, defined by the median vote, the formula for calculating final payoffs in a period is given by:

1 $$\pi_{i} \left( {x_{i} , x_{j \ne i, } t} \right) = \left( {1 - t} \right)x_{i} + t\mathop \sum \limits_{i = 1}^{n} \frac{1}{n}x_{i}$$

In this equation, ${\pi }_{i}$ denotes the final payoff of individual *i*, ${x}_{i}$ denotes the individual’s pre-tax earnings from production and ${x}_{j\ne i}$ other individual group members’ earnings.

The instructions carefully described the properties of the tax rate to the participants.It was clearly explained that redistribution is made by collecting a portion of the individual earnings from production and redistributing this amount back to group members. Subjects were informed that positive values of $t$ attenuate income inequalities, while negative values of $t$ amplify them.^16^

Some special cases illustrate the redistributive policies allowed by this mechanism. Egalitarian and maximin policies coincide at $t=1,$ when all participants receive the same payoff. Libertarian and meritocratic choices coincide at $t=0$, in which case everyone retains their income from production. Purely selfish behavior implies a vote for $t=-1$ by participants who perform above the group mean, and a vote for $t=1$ by participants who perform below the mean.^17^

#### Learning measurement and exit questionnaire

After the 10th and last period, participants ended the study with one final round of the production task. This round of the real-effort task was incentivized through the same piece rate as before, 10 ECU per completed entry; but, in this case, there was no redistribution. We included this additional performance measure to get an indication of the level of learning in the task, since a participant’s task performance across the 10 periods of the production and redistribution activity may be influenced both by learning, or by strategic responses to implemented redistribution policies.

We also administered an exit questionnaire. This comprised various questions about demographics and political orientation.

#### Conditions: no risk vs. risk

To explore the effects of introducing risk into the production context, the *Risk* condition added random variation in the individual performance payments in the 10 periods of Part 3. This was implemented by letting the computer randomly draw a productivity parameter in each period, separately for each participant. This parameter was equally likely to be any integer from 0 to 20. The number of ECU generated from the production task in a period by a group member in the Risk condition equaled the number of correct entries times this random productivity parameter. Part 3 was otherwise identical between conditions.

### Implementation and information

The experiment took place in English at the University of Zurich. We recruited 415 students from the University of Zurich and the Swiss Federal Institute of Technology using the software h-root (Bock, Baetge and Nicklisch, 2014). We conducted 17 sessions—16 sessions with 25 participants and one with 15—using the software z-Tree (Fischbacher, 2007). In total, 200 participants took part in the No Risk condition and 215 in the Risk condition. In each session, five randomly chosen men and five randomly chosen women were assigned to same-sex groups for Part 3, while the remaining participants were randomly grouped, independently of their sex. In total we have 18 all-female, 17 all-male, and 48 mixed groups. Table 1 presents the number of male- and female-majority groups by treatment.^18^

**Table 1:** Overview of experiment

	Subjects		Groups	
	Male	Female	Male majority	Female majority
No risk	107	93	25	15
Risk	111	104	25	18
Total	218	197	50	33

Participants received full instructions, which were also read aloud, for each part of the study at the onset of that part. They were informed that each part was independent, such that any decision taken in one part would not influence the course of events in other parts. We took several steps to clearly explain the instructions and procedures, particularly emphasizing the redistribution mechanism. Immediately after receiving detailed instructions about the mechanism, participants spent 3 min interacting with a calculation screen in which they could test the effect of different redistribution parameters for any hypothetical distribution of earnings among the five group members. This screen was also available when answering control questions. Participants also saw the same calculation screen for 60 s at the onset of each subsequent period, together with information from all prior periods about the five group members’ earnings from production and final earnings.

Each period concluded with feedback. In addition to the redistributive policy, participants saw a table indicating, for each group member in that period, the income generated from production, the member’s rank in the group, the net transfers and final earnings. A scrollable box also provided information on the redistribution policy, as well as each group member’s production and final earnings, for all previous periods.

In addition to a 10 CHF participation payment, participants were paid for all incentivized tasks, and for all 10 periods of the production and redistribution activity in Part 3. Earnings in ECU were converted to money at the rate of 50 ECU to 1 Swiss Franc (CHF). Participants earned, on average, 50.5 CHF (approximately 54 USD).

## Results

We first test whether we replicate the gender gaps in basic preferences widely observed in the literature. Next, we study whether we observe gender voting gaps in Part 3 that are consistent with women supporting more progressive redistributive policies. We then test whether our individual-level measures of preferences and expectations reproduce gender gaps widely documented in the literature and, if so, to what extent these gaps provide a basis for gender gaps in voting behavior. Finally, we examine to what extent gender gaps in policy preferences impact the policies implemented in groups with different gender majorities.

## Gender gaps in policy preferences

**Fig. 2:**
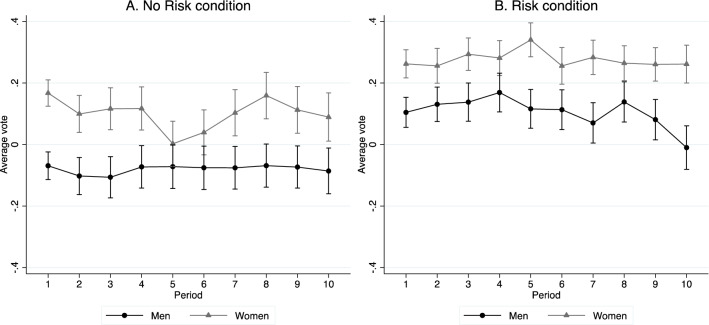
Mean vote across periods by gender and treatment

Recall that votes for the redistribution parameter, $t$, may range from −1 to 1, with higher values corresponding to more egalitarian policies. Figure 2 shows the average vote for the redistribution parameter, separately for men and women, across the 10 periods of Part 3. Panel A shows data from the No Risk condition, while Panel B shows the Risk condition.

A comparison of male and female votes in the first period supports the hypothesis that women favor more egalitarian redistribution. In Period 1 of the No Risk condition, men on average vote for negative redistribution coefficients $({\overline{t} }_{NR}^{ M}=-0.069)$, while women on average vote for egalitarian redistribution $({\overline{t} }_{NR}^{ F}=0.167)$ and the difference in means is statistically significant (${t}_{198}=3.78, p<0.001$). In the Risk condition, both men and women vote for more egalitarian redistribution relative to the No Risk condition, but men again vote for less egalitarian policies on average (${\overline{t} }_{R}^{ M}=0.105, {\overline{t} }_{R}^{ F}=0.262, {t}_{213}=2.348, p=0.020)$.^19^

These gender gaps in voting generally extend to subsequent periods. In both the No Risk and Risk conditions women vote, on average, for higher redistribution coefficients than men in all periods. While there is some variation over time in the average votes, there do not seem to be substantial and persistent time trends.

Table 2 analyzes voting patterns by gender using linear regressions. Models 1 and 2 study first-period votes, while Models 3 through 5 study voting across all 10 periods and include random effects at the subject level. All models cluster standard errors at the group level. The main observation from this table is that, across all specifications, the gender gap is sizable. Women vote for redistribution policies between 0.164 and 0.236 higher than those of men, and this gender gap is statistically significant ($p<0.002$ in all models).

**Table 2:** The gender gap in voting

Dependent variable:	Vote (Period 1)		Vote (All Periods)		
	(1)	(2)	(3)	(4)	(5)
Female	0.198*** (0.049)	0.236*** (0.058)	0.179*** (0.046)	0.181*** (0.058)	0.164*** (0.046)
Risk condition		0.173*** (0.064)		0.185*** (0.066)	
Female X Risk condition		-0.079 (0.094)		-0.010 (0.087)	
Period					-0.004 (0.004)
Female X Period					0.003 (0.007)
Constant	0.019 (0.035)	-0.069* (0.036)	0.014 (0.036)	-0.080** (0.039)	0.039 (0.038)
Observations (subjects)	415	415	4150	4150	4150
*R*-squared	0.042	0.064	0.028	0.056	0.028

Estimates from linear regressions. Models 1 and 2 use only data from the first period; Models 3 through 5 use votes in all 10 periods and include random effects at the subject level. Standard errors (clustered at the group level) in parentheses; *** *p* < 0.01, ** *p* < 0.05, * *p* < 0.1

Models 2 and 4 additionally include an indicator variable for the Risk condition and for its interaction with gender. The introduction of risk produces votes for more egalitarian redistribution policies—an impact roughly comparable to that of the gender gap. However, there is no significant interaction between the risk condition and gender, indicating that women are no more likely to favor more egalitarian redistribution in the presence of greater income risk. This provides an initial indication that the gender gap in voting may not be substantially influenced by differences in risk preferences between men and women. Finally, Model 5 confirms that there seems to be no substantial change across periods in the types of policies supported by men versus women, consistent with the lack of clear trends in Fig. 2.

### What drives gender gaps in policy preferences?

Having established that women tend to vote for redistribution policies that implement less risk and competition and more equality, we next investigate the extent to which this gap can be accounted for by gaps in more basic preferences. Since Table 2 reveals no interaction between gender and our experimental conditions in voting behavior, in most of our subsequent analysis we control for the Risk condition but omit interaction terms.

#### Gender preference gaps

Table 3 lists the individual-level measures elicited in Parts 1 and 2, with averages presented separately by gender. We replicate many of the gender gaps observed in earlier research. Men exhibit greater risk tolerance both in the incentivized investment task and in the survey question. The incentivized primary measure of Social Value Orientation, capturing the degree to which an individual puts positive weight on another’s payoff, shows women are only slightly more pro-socially oriented than men. On the secondary Social Value Orientation measure, however, which identifies a preference for efficiency over equality, we find that women are relatively more equality than efficiency oriented than men.^20^ Women further state a higher willingness to donate money in a non-incentivized survey question. Finally, men are also more willing to have their payment determined through a competitive incentive scheme.

**Table 3:** Gender gaps in preferences, expectations and task performance

Variable	Men	Women	Cohen’s *d*	*p*-value
Risk (Investment task) (incentivized 0–100, 100 = risky)	70.54 (2.02)	55.72 (1.88)	0.525	< 0.001
Risk (Survey question) (non-incentivized 0–10, 10 = risk taking)	6.10 (0.15)	5.03 (0.16)	0.481	< 0.001
Social Value Orientation (Primary) (Incentivized, -45 = competitive; 90 = altruistic)	17.12 (0.94)	18.26 (0.95)	-0.084	0.298
Social Value Orientation (Secondary) (Incentivized, 0 = egalitarian; 1 = efficiency)	0.67 (0.01)	0.61 (0.01)	0.408	< 0.001
Giving (Survey question) (non-incentivized, 0–1000, 1000 = generous)	135.55 (11.93)	202.34 (12.24)	-0.384	< 0.001
Competitiveness (0 or 1, 1 = competitive)	0.43 (0.03)	0.16 (0.03)	0.614	< 0.001
Relative performance beliefs (Part 2) (guessed rank: 0 = worst; 100 = best)	54.56 (1.17)	45.57 (1.16)	0.534	< 0.001
Average performance (Initial piece rate in Part 2)	12.05 (0.19)	11.61 (0.17)	0.164	0.188
Average performance (Final piece rate)	17.14 (0.24)	16.34 (0.20)	0.247	0.018^a^
Observations	218	197		

Standard error of the mean in parentheses. The table reports p-values from Wilcoxon Mann–Whitney tests. Cohen’s *d* is a standardized measure of the difference in means between two variables (the difference in means divided by the pooled standard deviation).

^a^ Since the data in the final piece-rate round are not independent, we also estimated a linear regression of performance on gender, clustering standard errors by group. This also yields a significant difference (*p *= 0.010).

The final three rows show performance in the task under piece rate incentives—in Part 2 and at the end of the experiment—along with beliefs about relative performance elicited in Part 2. We construct relative performance beliefs by converting the incentivized guess of relative rank in the session into a score from 0 (worst) to 100 (best). Men tend to believe that their Part 2 task performance ranks higher than do women, and this difference is statistically significant. This is consistent with earlier work documenting a gender gap in confidence.^21^ The second to last row in Table 3 presents the average actual performance on the real-effort production task in Part 2. We observe a slight, but statistically insignificant difference between male and female performance at this stage. The distributions of initial performance also do not differ significantly (*p* = 0.481, using a Kolmogorov–Smirnov test). However, importantly for our purposes, we find ample variation in initial task performance: the minimum performance is 5, the maximum performance is 23 and only 14 percent of observations lie at the median of 12. This is important, as such variation creates a potential motive for redistribution.

Table 3 additionally presents average performance in the final instance of the task, performed at the end of the experiment under piece-rate incentives. We find evidence of learning by both men and women—performance is considerably higher in this final measure than in the initial one, by 42 and 41 percent for men and women, respectively. We also find that the initially small male advantage is now larger and statistically significant in a Wilcoxon rank-sum test, although, the distributions of performance by gender at the end of the study do not differ significantly (*p* = 0.121, Kolmogorov–Smirnov test).^22^

#### Determinants of voting in Period 1

We next investigate our main question: to what extent do gender gaps in the individual characteristics in Table 3 account for the gender voting gap? We first focus on Period 1, where participants have no experience with the production and redistribution activity in their group.

Table 4 presents regressions with a participant’s first period vote as the dependent variable. In Model 1, we identify the overall gender effect on votes, with only the risk condition as a control; this replicates the pattern of findings from Table 2, where we observed both a gender gap and treatment effect on voting, which were largely independent of each other.

**Table 4:** The impact of preferences and performance beliefs on first-period votes

Dependent variable	Vote (Period 1)						
	(1)	(2)	(3)	(4)	(5)	(6)	(7)
Female	0.196*** (0.048)	0.177*** (0.049)	0.165*** (0.045)	0.161*** (0.050)	0.127*** (0.048)	0.122** (0.049)	0.080 (0.049)
Risk condition	0.136*** (0.049)	0.142*** (0.050)	0.125** (0.049)	0.135*** (0.048)	0.124*** (0.050)	0.105** (0.047)	0.097** (0.048)
Risk preferences (Investment task, standardized)		0.001 (0.024)			0.023 (0.024)		0.019 (0.024)
Risk preferences (Survey question, standardized)		-0.040 (0.027)			-0.034 (0.027)		-0.040 (0.026)
Social Value Orientation—Primary (competitive-altruistic, standardized)			0.067** (0.026)		0.069** (0.026)		0.067*** (0.025)
Social Value Orientation—Secondary (egalitarian-efficiency, standardized)			-0.056*** (0.021)		-0.052** (0.023)		-0.042* (0.022)
Giving (Survey question, standardized)			0.009 (0.023)		0.012 (0.024)		0.009 (0.024)
Competition (1 = competitive)				-0.129** (0.050)	-0.124** (0.049)		-0.050 (0.046)
Relative Part 2 performance beliefs (standardized)						-0.139*** (0.024)	-0.125 *** (0.023)
Part 2 task performance (standardized)						-0.015 (0.023)	-0.021 (0.023)
Constant	-0.050 (0.036)	-0.044 (0.034)	-0.029 (0.034)	0.006 (0.040)	0.026 (0.039)	0.001 (0.035)	0.040 (0.038)
Observations	415	415	415	415	415	415	415
R-squared	0.062	0.069	0.104	0.076	0.122	0.145	0.189

Estimates from linear regressions. Standard errors (clustered at the group level) in parentheses, *** *p* < 0.01, ** *p* < 0.05, * *p* < 0.1

Models 2 through 5 introduce the individual preference measures for risk, pro-sociality and competitiveness from Table 3, first separately for each domain and then jointly.^23^ For comparability, we standardize all preference measures, except for the binary measure of competitiveness. Model 2 introduces the two measures of risk-seeking, finding that neither has a statistically significant relationship with voting in the first period. Their introduction lowers the coefficient for gender slightly, by about 10 percent. Model 3 introduces the measures of pro-social concerns, finding that both Social Value Orientation measures predict voting in the expected directions—i.e., more altruistic concern and greater egalitarianism are associated with a preference for more positive redistribution coefficients—and both relationships are statistically significant. The unincentivized survey measure of pro-sociality has little relationship with voting. Introducing these measures of pro-social concern lowers the coefficient for female by about 15 percent. Finally, Model 4 shows that a preference for competition is associated with voting for lower redistribution coefficients and that this relationship is statistically significant. Introducing this preference measure lowers the gender coefficient by roughly 18 percent. Model 5 introduces all of the preference measures jointly; this lowers the coefficient for female by 35 percent, though the relationship between gender and voting remains statistically significant. This indicates that the elicited preference measures can account for part of the gender voting gap, though a large part of the gap remains unexplained.^24^

In Models 6 and 7, we introduce two measures of expected relative task performance. First, we introduce the incentivized measure of beliefs regarding relative task performance in Part 2, when participants completed the task under piece-rate incentives. This measure is negatively associated with the preferred redistribution coefficient, indicating that subjects who expect to perform (relatively) better on the decoding task vote for less egalitarian redistribution. We also include actual Part 2 task performance. This seems to have little predictive value in this model, though when included individually it also has a negative and statistically significant coefficient (see Online Appendix Table OA1). Introducing these measures of expected task performance lowers the coefficient for female by about 38 percent relative to Model 1, indicating that a substantial part of the gender gap in voting is driven by differential performance expectations.

Model 7 includes all preference and belief measures jointly. In this case, the coefficient for female is reduced by almost 60 percent and is no longer statistically significant ($p=0.11$). Further, the primary Social Value Orientation measure of altruistic concern is the only preference measure that remains significant, suggesting that relative performance beliefs may drive a large part of the impact on voting of the gender gap in competitiveness. Thus, combining our preference measures with measures of performance beliefs, the belief measures seem to account for a large part of the gender gap in voting, while preference measures have diminished explanatory power. Moreover, including all of these measures jointly substantially reduces the explanatory power of gender.^25^

#### Determinants of voting in Periods 2 through 10

We next investigate the degree to which basic preferences impact voting in the remaining periods of Part 3. This provides us with some insight into whether the influence of preference gaps persists through the process of repeated experience and learning.^26^

Table 5 reports regressions similar to those in Table 4, but using votes in Periods 2 through 10 as the dependent variable. Model 1 again reproduces the observation of a substantial gender gap in voting, along with a gap based on risk in the production task. Model 2 introduces the preference measures collected in Parts 1 and 2. This model is comparable to Model 5 in Table 4, but in contrast to the model for Period 1, where social preferences had significant explanatory power, only the coefficient for competitiveness remains statistically significant when predicting behavior in later periods. The introduction of the preference measures lowers the coefficient for gender by approximately 22 percent, which is smaller than the reduction of approximately 35 percent between Models 1 and 5 in Table 4.

**Table 5:** The impact of preferences and performance beliefs on votes in Periods 2–10

Dependent variable:	Vote (Periods 2–10)			
	(1)	(2)	(3)	(4)
Female	0.173*** (0.047)	0.135** (0.053)	0.085* (0.044)	0.069 (0.048)
Risk condition	0.186*** (0.041)	0.177*** (0.062)	0.160*** (0.041)	0.156*** (0.042)
Risk preferences (Investment task, standardized)		0.033 (0.032)		0.020 (0.028)
Risk preferences (Survey question, standardized)		-0.017 (0.029)		-0.018 (0.024)
Social Value Orientation—Primary (competitive-altruistic, standardized)		0.028 (0.031)		0.032 (0.027)
Social Value Orientation—Secondary (egalitarian-efficiency, standardized)		-0.021 (0.026)		-0.018 (0.023)
Giving (Survey question, standardized)		-0.009 (0.027)		-0.011 (0.023)
Competition (1 = competitive)		-0.150** (0.066)		-0.064 (0.055)
Relative Part 2 performance beliefs (standardized)			-0.120*** (0.024)	-0.111*** (0.024)
Lagged relative performance (standardized)			-0.172*** (0.016)	-0.172*** (0.015)
Constant	-0.081** (0.035)	-0.013 (0.043)	-0.026 (0.034)	0.003 (0.039)
Observations	3735	3735	3735	3735
*R*-squared	0.050	0.068	0.377	0.385

Estimates from linear regressions with random effects at the subject level. Robust standard errors in parentheses, *** *p* < 0.01, ** *p* < 0.05, * *p* < 0.1

Model 3 adds two measures of relative performance beliefs. First, we introduce the same measure of subjects’ beliefs from Part 2 that we used in Model 6 in Table 4. Second, we use the subjects’ lagged relative performance rank in the group as a proxy for beliefs about future relative performance. Both measures are standardized, with higher scores indicating higher (expected) performance. Both coefficients indicate that better expected performance is statistically significantly associated with votes for lower redistribution coefficients ($p<0.001$). Introducing these measures reduces the size of the gender coefficient by roughly 50 percent and this coefficient is now marginally statistically significant ($p=0.053$). Thus, as in Period 1, relative performance expectations seem to play at least as large a role in determining the gender gap in voting as our preference measures for risk, pro-sociality and competitiveness.

Finally, Model 4 combines all of the explanatory variables. The coefficients for the two measures of expected relative performance retain their magnitudes and statistical significance, but the coefficient for competitiveness is substantially smaller and no longer statistically significant, underlining the importance of performance beliefs for the gender gap in voting. In this model, the coefficient for gender is 60 percent smaller than in Model 1 and no longer statistically significant ($p=0.152$).^27^

To conclude the analysis of individual voting, our results suggest some impact of gender gaps in preferences for pro-sociality and competitiveness in accounting for the gender voting gap. However, a more important determinant of the gender voting gap appears to be differential performance expectations. In Period 1, perceptions of relative task ability account for 38 percent of the gender voting gap, while in Periods 2 through 10 they account for approximately one-half of this gap.

### Does gender composition impact group policy choices?

We now investigate the degree to which gender gaps in voting translate into differential outcomes in male- versus female-majority groups. Recall that individual votes were aggregated into collective outcomes through a median-voting rule.

**Fig. 3:**
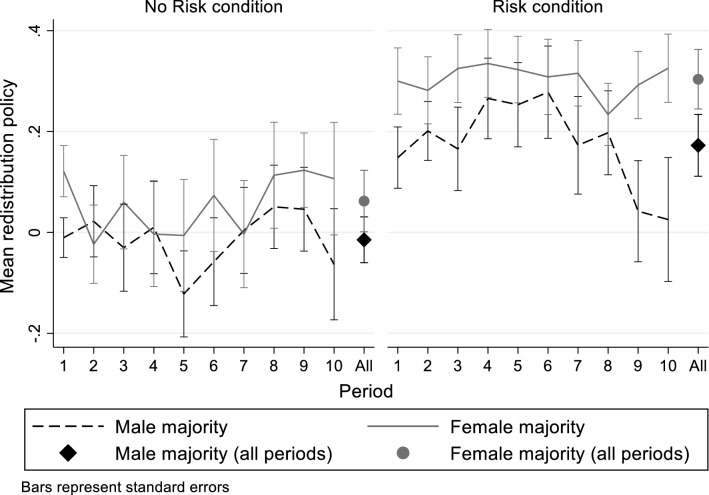
Average redistribution policy by group composition and treatment

Figure 3 presents the average policy implemented across the 10 periods of Part 3, separately for each condition and for male-majority and female-majority groups. The final set of markers for each condition shows the mean redistribution policy across all periods, using the average in a group across all periods as the unit of observation. Consistent with our observations of voting at the individual level, groups in the Risk condition implement more egalitarian redistribution policies than those in the No Risk condition.

In the first period, majority-female groups implement more egalitarian redistribution policies, relative to male-majority groups, both in the No Risk condition $$({\overline{t} }_{male maj}=-0.010; {\overline{t} }_{fem maj}=0.121$$; $${t}_{38}=2.053, p=0.047)$$ and in the Risk condition $$({\overline{t} }_{male maj}=0.148; {\overline{t} }_{fem maj}=0.300$$; $${t}_{41}=1.669, p=0.103)$$. Thus, groups in which women hold policy control tend to implement more progressive redistribution, but the gaps in outcomes are smaller than the mean voting gaps between men and women that we observed earlier.^28^ This naturally results from the central tendency of an aggregation mechanism such as median voting.

Over the remainder of the 10 periods, female-majority groups generally—though not always—implement more egalitarian redistributive policies than male-majority groups. Pooling across all periods, the average implemented redistribution policy is higher in female-majority groups in both the No Risk $({\overline{t} }_{male maj}=-0.015; {\overline{t} }_{fem maj}=0.062$) and Risk (${\overline{t} }_{male maj}=0.173; {\overline{t} }_{fem maj}=0.304)$ conditions. However, these differences in means are not statistically significant when using the average policy in a group across the 10 periods of Part 3 as an observation (No Risk: ${t}_{38}=1.020, p=0.314$; Risk: ${t}_{41}=1.492, p=0.144$).^29^

Table 6 presents regression analysis of the group policies implemented across the 10 periods of Part 2, using the redistribution policy in a group in a period as the dependent variable. The table follows a similar structure to our earlier analyses of individual voting (e.g., Table 4). Model 1 includes as explanatory variables whether the group had a male or female majority and the treatment condition. Groups with more women than men tend to implement more egalitarian redistribution policies, although the coefficient is only marginally statistically significant ($p=0.081$) and the magnitude of the difference (0.102) is roughly half as large as the gender gap in individual voting (cf. Model 1 in Tables 4 and 5).

**Table 6:** Implemented redistribution policies and group composition

Dependent variable:	Policy implemented in group (Periods 1–10)						
	(1)	(2)	(3)	(4)	(5)	(6)	(7)
Female majority	0.102* (0.058)	0.107* (0.064)	0.043 (0.064)	0.108* (0.058)	0.046 (0.064)	0.083 (0.055)	0.028 (0.061)
Risk condition	0.213*** (0.058)	0.209*** (0.060)	0.214*** (0.056)	0.207*** (0.059)	0.202*** (0.057)	0.175*** (0.060)	0.153** (0.060)
Median risk preference (Investment task, standardized)		0.027 (0.042)			0.005 (0.040)		0.050 (0.041)
Median risk preferences (Survey question, standardized)		-0.010 (0.044)			0.001 (0.046)		0.000 (0.043
Median SVO—Primary (competitive-altruistic, standardized)			-0.057 (0.042)		-0.051 (0.043)		-0.052 (0.042)
Median SVO—Secondary (egalitarian-efficiency, standardized)			-0.017 (0.053)		-0.032 (0.054)		0.014 (0.061)
Median Giving (Survey question, standardized)			0.143** (0.057)		0.161*** (0.059)		0.160*** (0.025)
Median Competition (1 = competitive)				0.087 (0.083)	0.137 (0.087)		0.146^*^ (0.079)
Median Relative Part 2 performance beliefs (standardized)						-0.074 (0.053)	-0.102* (0.060)
Median Part 2 task performance (standardized)						-0.028 (0.024)	-0.019 (0.024)
Constant	-0.027 (0.044)	-0.026 (0.045)	0.021 (0.047)	-0.038 (0.044)	0.015 (0.045)	0.325 (0.276)	0.257 (0.277)
Observations	830	830	830	830	830	830	415
R-squared	0.171	0.176	0.235	0.181	0.259	0.208	0.299

Estimates from linear regressions. Standard errors (clustered at the group level) in parentheses, *** *p* < 0.01, ** *p* < 0.05, * *p* < 0.1

The remaining models introduce measures of preferences in the group as explanatory variables, in a manner similar to that in our earlier analysis of individual voting behavior.

Since the critical voter in a group is the median voter, we use the median preference in a group as the explanatory variable to capture the potential influence of the most relevant preferences on group outcomes; this is analogous to our measure of the impact of gender, *Female majority*, which identifies whether the median gender is female. Looking at Models 2 through 5, the median risk preferences (Model 2) and competitiveness (Model 4) in a group do little to account for the policy gap between male- and female-majority groups. However, introducing measures of social preferences (Model 3) reduces the size of the gender majority coefficient by roughly 60 percent, with the median response to the hypothetical giving question providing the most explanatory power. Model 5, which introduces all of the preference measures jointly, shows that this social preference measure persists in having a statistically significant relationship with the group policy when simultaneously introducing the other measures of median group preferences. Models 6 and 7 additionally introduce the measures of expected task performance from Table 4—the median belief in the group about relative task performance in Part 2 and the median actual task performance in Part 2. The negative (though generally not statistically significant) coefficients indicate that groups in which the median expected or actual performance in Part 2 is higher also tend to adopt less egalitarian redistribution coefficients. Comparing Models 1 and 7, we see that accounting for the median preferences and performance expectations in a group accounts for a large proportion (approximately 70 percent) of the policy gap between male-majority and female-majority groups.

## Conclusion

We study the relationship between gender gaps in policy preferences and gaps in more basic preferences. There is widespread evidence that men and women differ in their attitudes toward risk, competition and inequality. Several studies also document that men and women sometimes exhibit different voting behavior, with women favoring greater redistribution. However, the degree to which gender gaps in the policy preferences of men and women are the direct result of more basic preference gaps—rather than of other factors, such as differential economic circumstances—requires better understanding.

To investigate this question, we design an experimental environment in which individuals repeatedly vote for redistribution policies and then engage in production subject to these policies. Consistent with evidence from outside the laboratory, women tend to vote for more egalitarian redistributive policies than men. This gap is substantial and persists with experience and is also very similar in environments with and without risk in the relationship between work and initial income.

We also replicate many previously observed gender gaps in more basic preferences. Women prefer less risk and less competition, prioritize equality over efficiency and report a greater willingness to share wealth. They are also less confident about their relative baseline performance in the task that we employ as the production activity, despite there being no gap in actual baseline performance. We then investigate the extent to which these gaps in basic preferences and expectations can account for the gender gap in voting. Our data suggest that preferences do play a role in voting behavior—particularly social preferences and competitiveness. However, differential expectations of future economic outcomes between men and women appear to have a larger impact on voting behavior. In combination, these two sets of factors go a long way in explaining the gender gap in policy preferences.

Finally, we also study whether the gender gap in policy preferences yields different policies enacted in groups where women, rather than men, hold the majority. We find this to be the case, but the magnitude and statistical strength of the group-level policy gaps is considerably smaller than the gaps at the individual level. Some of this naturally reflects a centralizing tendency of many social choice rules, including those like ours in which the median preferences have a large degree of impact.

Our work is important for better understanding how policies enacted in societies and organizations may change as women exert greater influence and control. First, our finding that expectations about relative performance appear to be a more important factor in explaining the gender gap in voting than gaps in more fundamental preferences indicates that the tendency for women to favor greater redistribution than men may diminish as women obtain better economic outcomes and security. Second, the relatively small policy gaps that we observe at the group level between male-dominated and female-dominated groups indicates that changes in policy outcomes from women exerting greater policy control may not be as dramatic as one might expect when extrapolating from average preference gaps at the individual level. Thus, claims that the world would be a fundamentally different place if women were to control policymaking should be tempered by the fact that such impacts may be relatively small. Our findings also provide an interpretation for why male- and female-majority groups often do not produce very different outcomes, despite the fact that gender differences in preferences seem quite reliable.

It is also worth noting that our evidence comes from contexts that we designed to create a straightforward relationship between the types of preferences often found to differ by gender and the unidimensional policy domain over which people vote. A natural open question is whether such differences persist in other contexts—for example, when the relationship between gender gaps in preferences for risk, competition and equality do not line up to predict concordant directional effects on policy preferences. Our work thus highlights the need for more careful study of precisely how gender differences scale up and persist over time to shape firms, institutions and societies.

## Supplementary Information

Below is the link to the electronic supplementary material.

Supplementary file1 (DOCX 232 kb)

Supplementary file2 (DOCX 97 kb)

## Footnotes

1 As an example, Christine Lagarde famously stated in 2010 that “If Lehman Brothers had been ‘Lehman Sisters,’ today’s economic crisis clearly would look quite different.” In a video interview published in 2019 by *The Economist*, former Liberian president Ellen Johnson Sirleaf stated, “If women were presidents all over the world, the world would be a safer place, it would be a more peaceful place, it would be a place of equality” (https://www.youtube.com/watch?v=PS9hlNhyRZY).

2 While the ultimate objective should be to explore these relationships in broader populations and with regard to real-world policy outcomes, laboratory experiments are a valuable starting point. A laboratory environment allows us to precisely measure individual ability, relative performance beliefs, as well as a variety of economic preferences, and document whether they differ by gender. The laboratory also allows us to create a situation in which basic preferences can plausibly directly translate into voting behavior, in contrast with non-laboratory settings involving complex policies that bundle many dimensions related to gender preference gaps in various and sometimes conflicting ways.

3 While there are many possible social choice rules that one could explore with our design, implementing the median voter’s preferences has several desirable properties (cf. Agranov and Palfrey, 2015). It is a simple and easy to understand voting mechanism, and one that can be implemented quickly in repeated periods. The median voter’s ideal point on a one-dimensional policy issue—the linear “tax rate” in our experiment—is also the outcome likely to arise under majority rule, per the median voter theorem.

4 This results mechanically from the fact that the joint median when sampling from two distributions will typically lie between the medians (or means) of those distributions. However, to our knowledge, this argument has not been previously applied to argue why the impact of varying gender composition on policy may be small. We illustrate this point in Figure OA1 in the Online Appendix, which presents a simulation exploring how varying differences in the mean preferences of men and women translate into differential outcomes when either men or women are in the majority. The gaps in policies at the group level are considerably smaller—roughly 40–50%—relative to mean gaps in individual-level preferences.

5 An earlier version of this paper, titled “Do Gender Preference Gaps Impact Policy Outcomes,” was structured around testing four conditions: (1) whether gender gaps in basic preferences are robust, (2) whether they translate into voting behavior, (3) whether such voting behavior translates into different policy outcomes in male- and female-majority groups and (4) whether these differences persist with repetition and feedback. Based on helpful suggestions from anonymous reviewers and the editor, we reorganized the current paper to start by documenting voting gaps (2) and their persistence with experience (4), then investigating the extent to which such gaps can be accounted for by gaps in more basic preferences (1) and finally exploring the extent to which such gaps robustly influence policy outcomes at the group level (3). The substantive results are unchanged.

6 Some experimental studies exploring whether male versus female managers adopt different incentives find contradictory results. Price (2011) finds male and female managers to be equally likely to implement tournament compensation as opposed to piece-rate incentives, but Shurchkov and van Geen (2019) find that men implement competitive incentives more often.

7 For a recent overview of this literature, see Hessami and da Fonseca (2020).

8 Exploring the extent to which typical gender gaps may persist past the glass ceiling, Adams and Funk (2011) survey directors and CEOs of publicly listed Swedish companies. While they find many similarities between male and female CEOs, they also find that female directors are less risk averse than their male counterparts, suggesting a reversal of the basic gender gap in preferences they find among a representative sample. Thus, while there is often apparent agreement that gender differences in some behavioral characteristics are robust and large, such gaps may be limited to a more specific set of contexts and conditions than sometimes suggested in the literature.

9 More precisely, in a model without controls for socio-economic status—but controlling for age, marital status, number of children, education and employment status—women exhibit more favorable attitudes toward redistribution, by 0.100 of a standard deviation (with a standard error of 0.059) and this coefficient is marginally statistically significant (*p* < 0.1). Adding controls for socio-economic status decreases the coefficient to 0.084 (with a standard error of 0.058) and the relationship is no longer statistically significant. These coefficients suggest a modest tendency for women to express more positive attitudes toward redistribution. Adding risk aversion as an explanatory variable decreases the gender coefficients to 0.085 (0.059) and 0.070 (0.059), respectively.

10 A recent study by Buser et al. (2020) conducts secondary analysis on data from two earlier experimental studies involving redistribution, conducted in eight university laboratories in four countries. They find that women demand more redistribution than men in only one of the eight locations (one of three in the US). Even then, this gender gap only arises in some choices (e.g., when income is based on performance rather than luck and when individuals have no information on their pre-tax income), but not in others (e.g., when individuals know their pre-tax income).

11 A related strand of literature explores whether the gender composition of teams impacts performance (for a review, see Azmat and Petrongolo (2014**)). For example, Apesteguia, Azmat and Irriberi (2012) use data on three-person teams performing in a large online business game. They find that all-female teams perform the worst, and this is explained by, among other things, all female teams implementing a less aggressive pricing strategy, and investing more in sustainability initiatives than other types of teams.

12 Complete instructions are provided in Appendix B.

13 As we describe in more detail below, we oversampled the assignment of men and women to single gender groups to increase variability of group composition. The number of such groups is too small to allow for a meaningful statistical analysis, and these groups are not analyzed separately.

14 The task was originally developed as part of the Wechsler Adult Intelligence Scale (Wechsler, 1958) and involves both memory and codification abilities and requires a mixture of effort and ability. We implemented this particular production task because we judged the task to have a neutral gender stereotype and because previous research shows the task to yield both no significant gender differences in performance and considerable variation in performance (Iriberri and Rey-Biel, 2011). While the first characteristic simplifies the analysis by not biasing the effect of the redistribution policy by gender, the second is necessary to create a motive for redistribution.

15 Incentivizing beliefs potentially creates a hedging motive. However, widespread hedging would suggest a negative relationship between actual rank and guessed rank, which we do not see in our data. Gächter and Renner (2010) find that incentives increase belief accuracy, leading us to opt in favor of incentivized beliefs.

16 The instructions also provided detailed examples in text and in tables of the impact of negative and positive redistribution coefficients, and illustrated the three special cases of $t$ = −1, 0 and 1. Finally, all participants also answered control questions consisting of numerical examples of both negative and positive redistribution policies.

17 When $t$ was negative, we limited its magnitude such that no group member would have negative earnings in a period. Specifically, the program adjusted $t$ upwards such that the least productive group member received a payoff of 0. This adjustment occurred rarely (in 4.6 percent of cases).

18 One all-male group and two all-female groups arose through random group assignment. There was a slight gender imbalance in our remaining population, as men attended the experiment more frequently. This yielded a substantially higher proportion of male-majority groups through random group assignment.

19 Online Appendix Figure OA2 shows the cumulative distributions of first-period votes by gender and condition; within each condition, the distribution of female votes first-order stochastically dominates that of male votes. For both conditions, the distributions of first-period votes also differ significantly in non-parametric Wilcoxon rank-sum tests (No Risk: $z=3.738, p<0.001$; Risk: $z=2.209, p=0.027$). Women also tend to vote substantially more frequently for policies that produce greater equality (i.e., $t>0$) than do men (No Risk: 33/107 (31%) for men versus 55/93 (59%) for women; Risk: 57/111 (51%) for men versus 74/104 (71 percent) for women).

20 The Social Value Orientation’s first six items yield a score between -45 and 90 degrees, representing the direction (degree) of an individual’s preferences on a two-dimensional space corresponding to own payoff (horizontal axis) and another’s payoff (vertical axis), yielding a spectrum from purely altruistic (90), to pro-social (45), individualistic (0) and competitive (-45). The remaining 9 items measure a tendency to prioritize equality (0) versus efficiency (1). The table reports the scores on the secondary measure for the entire sample of 415 participants. Restricting our analysis to the 160 individuals who expressed a pro-social orientation yields a similar gender gap (men: 0.64 (0.03), women: 0.54 (0.02); *d* = 0.408; *p* < 0.001).

21 We can directly measure overconfidence by comparing the rank provided by a subject to the actual performance rank. Men, on average, state a rank that is better than their actual rank (by 1.15), while women are slightly underconfident (−0.09). This difference is marginally statistically significant (*p* = 0.076, Wilcoxon rank-sum).

22 Figure OA3 in the Online Appendix provides data on performance across periods, separately for men and women. There is an apparent, though small, discontinuity in performance between Period 10 (with redistribution) and the final piece-rate performance measure (without redistribution). This suggests some strategic reduction of effort in response to the presence of redistribution.

23 Table OA1 in the Online Appendix presents regressions that introduce each measure separately. The two measures of risk preferences have the expected negative coefficients (i.e., risk seeking is negatively correlated with the preferred redistribution policy) but are statistically insignificant. The Primary SVO and the survey question on giving, which can be interpreted as measuring concern for others’ welfare, have positive coefficients, though only the Primary SVO is statistically significant (*p* < 0.01). The Secondary SVO, which measures emphasis on efficiency over equality, has a negative and statistically significant coefficient (*p* < 0.01). Finally, the two measures of performance and beliefs have negative and statistically significant (*p* < 0.02) coefficients when introduced separately. Online Appendix Table OA2 presents the raw correlations between first-period votes and each of the preference and belief measures, separately by gender and treatment.

24 In additional exploratory analysis (not reported here), we find that maternal and paternal education, number of siblings and age do not correlate with votes. However, participants who report being toward the right, rather than left, of the political spectrum vote for lower redistribution coefficients (No Risk: -0.031 (Right) vs. 0.111 (Left), * p* = 0.027; Risk: 0.081 (Right) vs. 0.260 (Left), * p* = 0.009, using a median split of self-reported political orientation), providing suggestive evidence that voting in our experiment may be related to political preferences outside the laboratory.

25 We also estimated separate versions of Model 7 for men and for women (see Online Appendix Table OA1), to study whether the relationship between our explanatory variables and the dependent variable differs by gender. These regressions indicate that performance beliefs significantly predict the chosen redistribution coefficient for both genders ($p<0.01$). For women, but not for men, the primary Social Value Orientation is also a significant predictor ($p=0.001$).

26 While there is variation in individual voting behavior across periods, we also find substantial consistency over time. For example, there is a strong positive correlation (0.82) between average votes in Periods 1–5 and in Periods 6–10 (see Online Appendix Figure OA4). This consistency may be partly explained by the high positive correlation of 0.70 between the individual performance rankings from one period to the next throughout the experiment.

27 We also estimated versions of Model 4, separately, for the No Risk and Risk conditions (see Online Appendix Table OA3). The only substantive difference is that the coefficient for lagged relative performance is roughly twice as large in magnitude in the No Risk condition (-0.231 (0.024)) than in the Risk condition (−0.120 (0.018)). Table OA3 also reports separate versions of Model 4 for each set of three periods (2–4, 5–7, 8–10). The results are generally similar to those in Table 4; perhaps the most notable difference is that the coefficient for competitiveness is marginally statistically significant in Periods 1–3 ($p=0.069$).

28 Recall that our design oversamples groups with extreme (all-male, all-female) gender compositions. This means that the policy gaps between male- and female-majority groups might be exaggerated relative to what would arise if group gender composition were determined by forming groups at random from the entire population. Our first-period data, in which group members have no prior feedback from any interactions with other participants, provide insight into what kind of gaps would arise between male-majority and female-majority groups if they had been formed at random. For each condition, we conducted 10,000 simulations in which we formed groups by randomly sampling individuals from the entire population within that condition, recording whether the group had a male or female majority, and implementing as “policy” the median Period 1 vote in the artificial group. In No Risk, the mean policy enacted in male-majority groups is 0.010 and for female-majority groups it is 0.096; the gap of 0.085 is smaller than the actual first-period gap in our experiment (0.132). In Risk, the mean policies are 0.176 (male-majority) and 0.244 (female-majority), and the gap of 0.067 is again smaller than the actual first-period gap we observed (0.152).

29 We also investigated differences between male-majority and female-majority groups in other outcome measures. For example, across the 10 periods of Part 3, male-majority groups generated more completed tasks per period than female-majority groups in the No Risk condition (male majority: 69.24; female majority: 63.72; t_38 = 3.342; *p* < 0.01), but not in the Risk condition (male majority: 63.62; female majority: 64.58; t_41=0.764; *p* = 0.450). At least part of the difference in the No Risk condition appears to be driven by initial differences in task ability. We observe no significant differences between male-majority and female-majority groups in the resulting degrees of inequality, measured by group-level Gini coefficients of final individual payoffs from Part 3.
