# Supplementary material for: Gender preference gaps and voting for redistribution
Source: Exp Econ. 2022 Jan 6;25(3):845–75. doi: 10.1007/s10683-021-09741-8 (PMC9165268; doi:10.1007/s10683-021-09741-8)
Supplement: Supplementary file 1 — Supplementary file1 (DOCX 232 kb) [file 10683_2021_9741_MOESM1_ESM.docx]

Online Appendix Ranehill Weber 2021

Table OA1: Relationships between preferences and performance beliefs on first-period votes

| *Dependent variable:* | *Vote in Period 1* | | | | | | | | | |
| --- | --- | --- | --- | --- | --- | --- | --- | --- | --- | --- |
|  | *All* | | | | | | | | *Male* | *Female* |
|  | (1) | (2) | (3) | (4) | (5) | (6) | (7) | (8) | (9) | (10) |
| Female | 0.190^***^  (0.049) | 0.177^***^  (0.048) | 0.189^***^  (0.047) | 0.166^***^  (0.047) | 0.183^***^  (0.047) | 0.161^***^  (0.050) | 0.122^**^  (0.049) | 0.186^***^  (0.049) |  |  |
| Risk condition | 0.136^***^  (0.049) | 0.142^***^  (0.049) | 0.123^**^  (0.049) | 0.136^***^  (0.048) | 0.133^***^  (0.048) | 0.135^***^  (0.048) | 0.105^**^  (0.047) | 0.132^***^  (0.048) | 0.116^*^  (0.069) | 0.072  (0.067) |
| Risk preferences  (Investment task, standardized) | -0.011  (0.023) |  |  |  |  |  |  |  | 0.007  (0.038) | 0.013  (0.036) |
| Risk preferences  (Survey question, standardized) |  | -0.040  (0.026) |  |  |  |  |  |  | -0.056  (0.038) | -0.021  (0.037) |
| Social Value Orientation - Primary  (competitive-altruistic, standardized) |  |  | 0.083^***^  (0.025) |  |  |  |  |  | 0.024  (0.044) | 0.107^***^  (0.032) |
| Social Value Orientation - Secondary  (egalitarian-efficiency, standardized) |  |  |  | -0.075^***^  (0.019) |  |  |  |  | -0.018  (0.033) | -0.060  (0.037) |
| Giving  (Survey question, standardized) |  |  |  |  | 0.033  (0.022) |  |  |  | 0.037  (0.030) | -0.008  (0.037) |
| Competition  (1 = competitive) |  |  |  |  |  | -0.129^***^  (0.050) |  |  | -0.022  (0.067) | -0.067  (0.090) |
| Relative performance beliefs  (standardized) |  |  |  |  |  |  | -0.144^***^  (0.024) |  | -0.135^***^  (0.036) | -0.112^***^  (0.034) |
| Part 2 task performance (standardized) |  |  |  |  |  |  |  | -0.058^**^  (0.023) | -0.045  (0.028) | 0.019  (0.031) |
| Constant | -0.049  (0.035) | -0.044  (0.035) | -0.040  (0.035) | -0.036  (0.035) | -0.042  (0.035) | 0.006  (0.040) | 0.001  (0.035) | -0.043  (0.035) | 0.028  (0.046) | 0.144^***^  (0.051) |
| Observations | 415 | 415 | 415 | 415 | 415 | 415 | 415 | 415 | 218 | 197 |
| R-squared | 0.063 | 0.069 | 0.091 | 0.085 | 0.067 | 0.076 | 0.144 | 0.077 | 0.158 | 0.185 |

Estimates from linear regressions. Standard errors (clustered at the group level) in parentheses, ^***^ p<0.01, ^**^ p<0.05, ^*^ p<0.1.

**Table OA2: Correlations between vote in Period 1 and individual characteristics**

|  | No Risk | | | Risk | | |
| --- | --- | --- | --- | --- | --- | --- |
|  | All | Male | Female | All | Male | Female |
| Risk (Investment task)  (incentivized 0-100, 100 = risky) | *-0.143*  *(0.043)* | -0.147  (0.130) | -0.020  (0.852) | -0.011  (0.877) | 0.057  (0.556) | 0.018  (0.860) |
| Risk (Survey question)  (non-incentivized 0-10, 10 = risk taking) | **-0.215**  **(0.002)** | **-0.297**  **(0.002)** | 0.006  (0.956) | -0.051  (0.461) | 0.050  (0.603) | -0.092  (0.353) |
| Social Value Orientation (Primary)  (Incentivized, -45 = competitive; 90 = altruistic) | *0.181*  *(0.011)* | 0.062  (0.524) | **0.293**  **(0.004)** | **0.181**  **(0.008)** | 0.048  (0.614) | **0.347**  **(<0.001)** |
| Social Value Orientation (Secondary)  (Incentivized, 0 = egalitarian; 1 = efficiency) | **-0.243**  **(0.001**) | -0.169  (0.082) | *-0.239*  *(0.021)* | *-0.161*  *(0.018)* | -0.024  (0.803) | **-0.264**  **(0.007)** |
| Giving (Survey question)  (non-incentivized, 0-1000, 1000 = generous) | 0.104  (0.142) | 0.038  (0.701) | 0.015  (0.886) | 0.108  (0.115) | 0.115  (0.231) | 0.066  (0.504) |
| Competitiveness  (0 or 1, 1 = competitive) | **-0.232**  **(0.001)** | *-0.239*  *(0.013)* | -0.033  (0.757) | -0.124  (0.070) | -0.068  (0.478) | -0.111  (0.263) |
| Relative performance beliefs  (guessed rank: 0 = worst; 100 = best) | **-0.394**  **(<0.001)** | **-0.480**  **(<0.001)** | -0.161  (0.122) | **-0.283**  **(<0.001)** | *-0.194*  *(0.041)* | **-0.337**  **(0.001)** |
| Average performance  (Initial piece rate in Part 2) | *-0.171*  *(0.015)* | -0.178  (0.067) | -0.060  (0.567) | -0.108  (0.114) | -0.159  (0.095) | -0.050  (0.618) |
| Observations | 200 | 107 | 93 | 215 | 111 | 104 |

Each cell reports the correlation between the corresponding individual-level measure and Period 1 vote. *Italics*: p<0.05; **bold**: p<0.01.

**Table OA3: The impact of preferences and performance beliefs on votes in Periods 2-10**

| *Dependent variable:* | *Vote* | | | | |  |
| --- | --- | --- | --- | --- | --- | --- |
|  | *Periods* | | | *Periods 2-10* | | |
|  | *2-4* | *5-7* | *8-10* | *No Risk* | *Risk* | |
|  | (1) | (2) | (3) | (4) | (5) | |
| Female | 0.052  (0.048) | 0.055  (0.055) | 0.092  (0.057) | 0.060  (0.061) | 0.096  (0.072) | |
| Risk condition | 0.187^***^  (0.044) | 0.171^***^  (0.048) | 0.117^***^  (0.048) |  |  | |
| Risk preferences  (Investment task, standardized) | 0.005  (0.025) | 0.042  (0.034) | 0.010  (0.031) | -0.014  (0.037) | 0.059  (0.037) | |
| Risk preferences  (Survey question, standardized) | -0.012  (0.022) | -0.021  (0.029) | -0.020  (0.027) | -0.003  (0.031) | -0.033  (0.037) | |
| Social Value Orientation - Primary  (competitive-altruistic, standardized) | 0.014  (0.023) | 0.027  (0.033) | 0.060^*^  (0.033) | 0.049  (0.045) | 0.028  (0.034) | |
| Social Value Orientation - Secondary  (egalitarian-efficiency, standardized) | -0.026  (0.024) | -0.011  (0.028) | -0.022  (0.024) | 0.006  (0.034) | -0.026  (0.028) | |
| Giving  (Survey question, standardized) | 0.002  (0.021) | 0.013  (0.028) | -0.048^*^  (0.028) | -0.056^*^  (0.023) | 0.021  (0.030) | |
| Competition  (1 = competitive) | -0.091^*^  (0.050) | -0.059  (0.064) | -0.041  (0.062) | -0.072  (0.075) | -0.069  (0.075) | |
| Relative Part 2 performance beliefs  (standardized) | -0.074^***^  (0.022) | -0.121^***^  (0.027) | -0.108^***^  (0.028) | -0.123^***^  (0.035) | -0.099^***^  (0.030) | |
| Lagged relative performance  (standardized) | -0.228^***^  (0.021) | -0.178^***^  (0.022) | -0.219^***^  (0.023) | -0.231^***^  (0.024) | -0.120^***^  (0.018) | |
| Constant | 0.014  (0.041) | -0.005  (0.045) | 0.000  (0.043) | 0.008  (0.048) | 0.145^**^  (0.058) | |
| Observations | 1,245 | 1,245 | 1,245 | 1,800 | 1,935 | |
| R-squared | 0.384 | 0.331 | 0.378 | 0.526 | 0.267 | |

Estimates from linear regressions with random effects at the subject level. Robust standard errors in parentheses, ^***^ p<0.01, ^**^ p<0.05, ^*^ p<0.1.

Figure OA1: An example of policy gaps as a function of individual preference gaps

In the simulation analysis presented above, we assume that men’s preferences are distributed according to a normal distribution, $v^{m}\sim N(0,1)$, and that women’s preferences are distributed similarly but with a different mean, $v^{f}\sim N(\theta,1)$. Our simulations construct five-person groups at random, classify them as majority male or majority female, and then identify the group’s median preference. This simple stylized example of a group making a collective choice parallels our experiment’s design. In the graph, the horizontal axis shows the mean gender preference gap, $\theta$, while the vertical axis presents the mean difference in implemented policies between majority-male and majority-female groups

**Figure OA2: Cumulative distributions of first-period votes by gender and condition**

**Figure OA3. Performance in task over time**

**
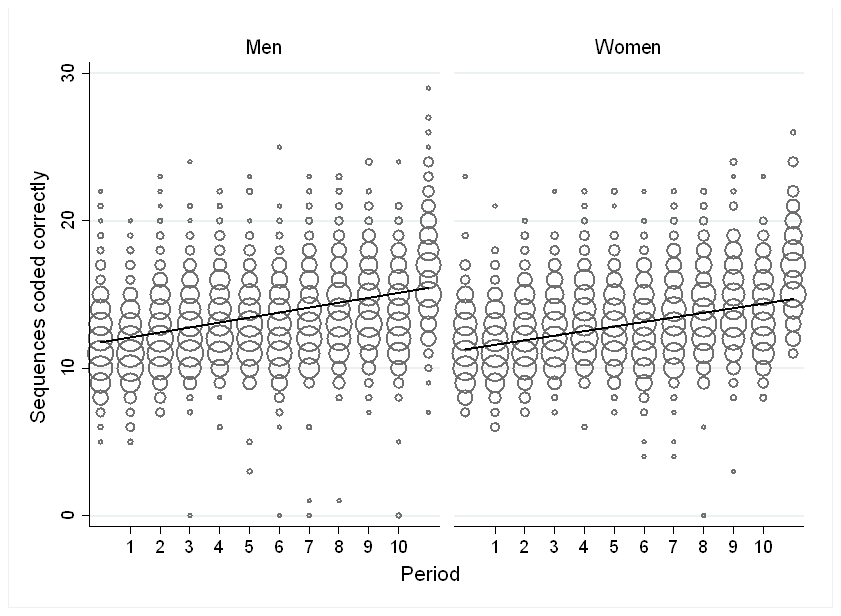
**

**Figure A4. Comparison of votes (first vs. second half of Stage 3)**
