# Supplementary material for: Gender preference gaps and voting for redistribution
Source: Exp Econ. 2022 Jan 6;25(3):845–75. doi: 10.1007/s10683-021-09741-8 (PMC9165268; doi:10.1007/s10683-021-09741-8)
Supplement: Supplementary file 2 — Supplementary file2 (DOCX 97 kb) [file 10683_2021_9741_MOESM2_ESM.docx]

**Appendix B : Instructions**

Initial Instructions

The experiment comprises three parts. We will provide you with detailed instructions before each part.

In addition to the CHF 10 payment that you receive for your participation, you will be paid an additional amount of money that you accumulate from decision tasks in the three parts of the study. The exact amount you receive will be determined during the experiment, and will depend on your decisions, and the decisions of others. Please note that the decisions you make in any part of the experiment will have no effect on what happens in other parts.

All monetary amounts you will see in this experiment will be denominated in experimental currency units (ECU). At the end of the experiment, your earnings in ECU will be exchanged into CHF at a rate of

**50 ECU = 1 CHF**

Note that all your interactions in the study are anonymous. This means that you will not know the identity of any other participant with whom you interact and no other participants will know your identity.

If you have any questions during the experiment, please raise your hand and wait for an experimenter to come to you. *Please do not talk, exclaim, or try to communicate with other participants during the experiment. Participants intentionally violating the rules may be asked to leave the experiment with only their participation payment.*

Please click “Continue” now to see the instructions for the first part of the study.

Instructions to Part 1

In Part 1 you will make a few simple economic decisions and answer some questions. You will receive detailed instructions before each decision.

Please note that each of the decisions you make in this part is independent and does not influence the future course of the study. For each decision you will be informed about the outcome, and the earnings you received, at the end of the study.

Please press the ''Continue'' button now to see the instructions for the first decision.

**Investment Decision**

The first decision is an investment decision. This decision is for real money; the result of your decision will be added to your account and paid to you at the end of the experiment.

You start the investment task with a balance of 100 ECU. You choose how much of this amount (from 0 ECU to 100 ECU) you wish to allocate to the investment.

The ECU that you choose *not to invest* will be saved in your account and cannot be lost. You will receive these ECU at the end of the experiment.

The value of the ECU you choose *to invest* depends on the success or failure of the investment.

The success or failure of the investment will be determined by a computerized random draw, similar to a coin flip. There are two possible outcomes:

- With 50% probability the investment fails and you lose the amount invested.
- With 50% probability the investment succeeds and you receive 2.5 times the amount invested.

So, for any amount X that you invest, you will keep 100 – X, regardless of what happens with the investment. If the investment fails, which happens with 50% probability, your earnings from this decision will be **100 – X**, since you lose the amount that you invested. If the investment succeeds, which also happens with 50% probability, your earnings from the decision will be 100 – X + 2.5*X = **100 + 1.5*X**

Please enter the amount you wish to invest on your screen now. If you have any questions about the investment task please raise your hand and an experimenter will come to you.

Your Decision:

Please enter the amount of money (from ECU 0 to ECU 100) you wish to invest:

ECU ______

Once you enter a number, please submit your investment by clicking "Continue".

If you have any questions about the investment task please raise your hand and an experimenter will come to you.

**Allocation Decisions**

In the second task, you will make 15 decisions in which you allocate ECU between yourself and an anonymous other participant. You are again making decisions for real money; the payoff from this task will be added to your account and paid to you at the end of the experiment. Any money you allocate to another participant will be paid to another randomly selected participant at the end of the experiment.

For each of the 15 decisions, you will see a range of possible allocations. Your task in each decision is to choose your preferred allocation among the alternatives.

After you have made your allocation decisions, one randomly chosen decision (among the 15) will be chosen for each participant and implemented. This means that you will receive payment for two randomly chosen decisions. This is because each decision involves a decision-maker and a receiver. You will be paid once for a randomly selected decision in which you chose, and once as a receiver for the allocation chosen by another participant. The participant you interact with in these two cases will not be the same. That is, the receiver from your implemented decision will not be the decision-maker in the decision for which you are the receiver.

Below you see a sample of what your screen will look like for each of the 15 allocation decisions. The numbers in this example are used only to illustrate the task and do not correspond to the numbers in the actual decisions. In the upper row, you will see the allocation for you, and in the bottom row the allocation for the randomly chosen receiver. In the example below, if you choose the leftmost allocation, you receive 1 and the other participant receives 9. If you choose the rightmost allocation, you receive 9 and the other participant receives 1. Notice that each time you select an allocation the corresponding payments for you and the receiver will be displayed to the right of the table.


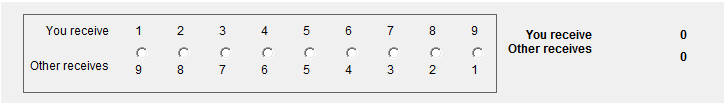


You will see 15 decisions, with varying allocations for you and another randomly selected participant. In each case, click on the button corresponding to the decision you would like to implement.

Once you are ready, please start the task by clicking "Continue".

If you have any questions about the allocation task please raise your hand and an experimenter will come to you.

**Questionnaire**

Before Part 1 ends, we would like you to answer a few additional questions. Please fill in your answers to the questions on the screen. If you have any questions raise your hand and an experimenter will come to you.

You confirm your entries by clicking the "Continue" button. Once everybody has answered these questions, Part 2 will begin.

1. First, state, in general, how willing or unwilling you are to take risks on a scale from 0 to 10. 0 means you are "completely unwilling to take risks" and a 10 means you are "very willing to take risks". You can also use any integer number between 0 and 10 to indicate where you fall on the scale. Possible choices are: 0, 1, 2, 3, 4, 5, 6, 7, 8, 9, 10.
2. Second, imagine the following situation: Today you unexpectedly received CHF 1,000. How much of this amount would you donate to a good cause? Values between 0 and 1,000 are allowed for the donation.
3. How old are you?
4. Are you male of female?

Instructions to Part 2

In Part 2 you will work on a task for 90 seconds. You will be paid, as explained in more detail below, based on your performance on the task. You will also make a few decisions in which you can make additional money. Your actions in Part 2 are independent of the other parts of the study, and do not in any way influence the future course of the study.

**The Production Task**

The task for Part 2 consists of a coding task. During the task, your screen will display a key, which consists of a series of unique matches between letters and numbers. More precisely, each key will show the numbers 1-9, and a series of 9 letters of the alphabet, displayed such that one letter corresponds to one number. An example of such a key is in the picture below. In this example, M corresponds to 1, U to 2, O to 3, and so on.


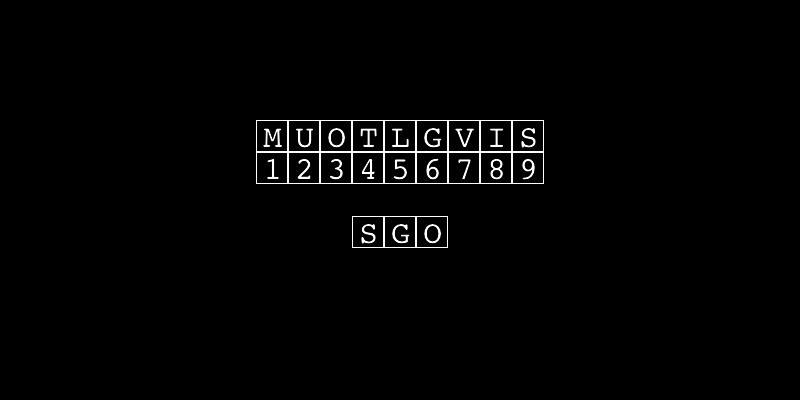


During the task, you will be shown sequences of three letters. Your task is to enter the corresponding three numbers on your computer. For example: if the sequence you are asked to code is SGO, as indicated in the picture, the correct answer for the key above is 963. You obtain this sequence of numbers by exchanging the S for a 9, the G for a 6, and the O for a 3.

The sequences of three letters will appear on your screen, one at a time. Once you enter the corresponding three digits, you confirm your answer with the “OK” button. If the entered sequence is correct, a new sequence will appear on your screen, otherwise you will be asked to recode the three-digit sequence until it is entered correctly. For each key of letters and digits, you will see nine three-digit sequences to code into numbers. Then a new key appears, for which you will get nine consecutive three-digit codes, and so on. You will have 90 seconds to enter as many sequences as you can.

Your payment from the task depends on the number of sequences you code correctly. In particular, you will be paid 10 ECU per correctly coded sequence. At the end of the experiment, you will be informed about your resulting earnings.

On the next screen, you will see an example screen similar to the one you will see during the actual task. In the upper half of the screen you will see a picture of the key, consisting of nine letter-number pairs. Below that you will see the sequence of three letters that you are to recode into a three-digit number, as well as the box where you enter your answer. Remember that you will receive 10 ECU for each sequence that you correctly type in during the 90 seconds.

Please click the “Continue” button on your screen now. You may then try the task on the example screen by filling in the correct three-digit number in the empty box. If the cursor is not positioned within the box, you will need to click inside the box to allow you to type in the box. Confirm your entry with the “OK” button. You will then be able to click to start the actual task for Part 2.

If you have any questions about the task please raise your hand and an experimenter will come to you.

**Part 2 – Additional Decisions**

To conclude Part 2, we would like to ask you a few questions related to the task you just completed.

First, we offer you the possibility to get paid once more for your performance in the task you just completed. This payment will be paid to you at the end of the experiment, in addition to all other payments, including the one you already earned for performing the task.

Specifically, we here ask you to make a choice regarding how you would like to be paid. In particular, you can choose between two different payment methods, method 1 and method 2.

Payment method 1

The first method is a certain payment of 10 ECU per correctly entered three-digit sequence. This method is the same as the payment you already received for performing the task. If you choose this payment method, you will receive an additional payment of the same size as before. So, under payment method 1, your payment depends only on how many sequences you entered correctly.

Payment method 2

If you choose the second method, your performance will be compared to the performance of a randomly chosen participant present here today, which could be any other participant in the room. You will be paid 20 ECU per correctly entered sequence, *only if you entered more three-digit sequences than this randomly chosen participant*. If you entered fewer sequences than this randomly chosen person you get nothing. If you entered equally many sequences, then the computer will randomly decide, with equal probability, whether you receive 20 ECU per correctly entered sequence, or whether you receive 0 ECU. So, under payment method 2, your payment depends partly on whether you entered more or fewer sequences correctly than another randomly selected participant.

Please indicate your choice by clicking on the payment method according to which you would like to get paid. You confirm your choice by clicking “Continue.”

If you have any questions about the task please raise your hand and an experimenter will come to you.

**Part 2 – Additional Decisions**

Finally, we ask you to assess your performance (how many three-digit sequences you coded correctly) during the 90-second task, in comparison to the performance of the other participants in the room. Please enter your guess of your performance ranking in the box below. There are *[Number of participants]* participants present today. If you, for example, think that you copied the most sequences, and that no other participant copied as many, you enter the rank of 1. If you think that 6 participants performed better than you, you enter the rank 7. If you think you copied the fewest sequences, and that all other participants copied more than you, you enter the rank [*Number of participants*]. You submit your guessed rank by clicking the "Continue" button.

Please consider your guess well. You will earn an additional 50 ECU if your answer lies within 2 ranks of the true one. That is, if your true ranking is only 0, 1, or 2 away from the rank that you guess, you will receive an extra payment of 50 ECU at the end of the study.

If you have any questions about the task please raise your hand and an experimenter will come to you. Otherwise, please enter your guess below.

My estimated rank: ___________________

Instructions to Part 3

Part 3 mainly comprises 10 periods of the decision-task described below. After the 10 periods, you will perform the same 90-second task as in Part 2 once more. We will then conclude the study with a short questionnaire.

At the beginning of Part 3, you will be randomly assigned to a group of 5 participants. For the remainder of the experiment, you will interact only with the other 4 participants in your group.

Each participant in a group will be randomly assigned a unique ID (A, B, C, D, or E). A participant’s ID remains constant for the entire study and allows you to follow other group members across different periods. Participants will be identified only by ID number, meaning that you will not know the identity of any other participant with whom you interact and no other participants will know your identity.

The course of each of the 10 periods in Part 3 is the same. Each period contains 3 steps. Below, we give you an overview of these steps. We then explain each step in detail.

1. The vote: In each period the participants may generate money. As a first step in a period the five group members will vote how they intend to allocate the money the group members generate in that period. The result of the vote is presented to all group members directly after the vote.
2. The production task: As a second step, participants take part in the production task, in which each participant can generate ECU. This is the same as the task from Part 2.
3. Display of final earnings: After everyone has performed the task, the computer reallocates the money produced from the task among the group members, based on the outcome of the vote at the beginning of that period.

Below we will explain each step of a period in detail.

**The production task [No Risk Condition]**

In each period of Part 3, each group member may work on the same production task as in Part 2, coding sequences of three letters into digits for 90 seconds and generating 10 ECU per correctly coded sequence. However, a group member’s final earnings in a period may differ from the money that person generates in the production task. How the final earnings differ from the money generated in the production task depends on the outcome of the group vote.

**The production task [Risk Condition]**

In each period of Part 3, each group member may work on the same production task as in Part 2, coding sequences of three letters into digits for 90 seconds and generating ECU.

Unlike in Part 2, the productivity values will now vary randomly between group members and periods. Specifically, in each period, the computer will randomly draw a new productivity value, with equal probability, from all integer values between 0 and 20, for each group member. This means that your productivity value in a period is equally likely to be any value from 0, 1, 2, . . ., 19 or 20. The ECU that you generate from the production task in a period will be the number of sequences you correctly code, multiplied by the randomly-determined productivity value.

Since your productivity value in a period is randomly drawn, it does not depend on the productivity value of any other participant, any previous productivity values, or any previous performance or decisions made in the experiment. Each member of a group may have a different productivity value. You will not know the value of your productivity value, or the values for anyone in your group, until the end of the period.

In Part 3, the ECU generated from the production task in a period differs between group members based on two things; the number of sequences that member codes correctly and that group member’s randomly determined productivity value.

A group member’s final earnings in a period may differ from the money that person generates in the production task. How the final earnings differ from the money generated in the production task depends on the outcome of the group vote.

**The vote**

At the beginning of each period, before participating in the production task, the members of a group will vote on a rule for reallocating the money generated by each of the five group members in the production task. This vote is taken before knowing the exact amounts of money generated by each group member in the task.

Specifically, group members must vote on a transfer parameter, **t**. Via their vote on t, the group members have the possibility to influence the final distribution of earnings among the group members in a period.

The final earnings in a period are determined by collecting a share of the earnings produced by each group member and by then redistributing this collected amount among the group members. The amount collected from a member may differ from the transfer the member gets back.

The transfer parameter, t, indicates how large the effect is on the individual earnings. Specifically, t has two effects:

1. First, the distance of t from zero indicates how much redistribution takes place. When **t = 0**, then all group members keep the money they generated in the production task and no redistribution takes place. As t gets farther from zero, a larger share of the group’s total production is collected for redistribution. The values of t can range from -1.00 to 1.00.
2. Second, t can be either positive, meaning it is greater than zero, or negative, meaning it is lower than zero. When **t is positive**, this means that the redistribution primarily collects money from those who generated more money in the production task and rewards those who generated less money in the production task. When **t is negative**, this means that the redistribution primarily rewards those who generated more money in the task and collects money from those who produced less in the production task.

We will explain in detail below what different values of t imply for the final payoffs, and illustrate it with a few examples.

At the beginning of a period, all group members vote for t by stating their preferred value. Each group member can propose any value of t, with a precision of two decimals, between -1.00 and 1.00.

Once the votes are cast, the group members’ 5 proposed values for t are compared, and the median value is implemented for the group in the relevant period. The median is the value that lies in the middle when the five votes are ordered from highest to lowest. In each period, a new vote is taken and **t takes the value of the median vote** for that period. Thus, depending on the votes by the group members, t may take a new value in each period.

Note that using the median vote means that each participant does best by specifying the true value of t that they prefer. That is, there is no reason to try to strategically manipulate your vote. Your vote cannot affect the value of t, unless it lies in the middle of all the votes and in that case your vote determines t for that period.

**Final earnings: The transfer parameter t**

The transfer parameter allows the group members to influence the final earnings in a period. Depending on the value of t, groups may choose to either compensate group members who generate less money in the production task (who coded fewer sequences) or to reward group members who generate more money. In particular,

- if the value of t is positive, group members who generated *less* money than the group average end up benefitting from the redistribution;
- if the value of t is negative, group members who produced *more* money than the group average end up benefitting from the redistribution.

We will now provide you with the formula that determines how t affects the earnings of group members. However, if you do not understand the formula in detail, do not worry. The key facts to remember are that i) the farther t is from zero the more redistribution takes place and ii) a positive value of t benefits those who generated less money than the group average while a negative value of t benefits those who generated more money than the group average.

Let *x_i_* denote the money generated by participant i in the production task, and t the transfer parameter. Remember that there are 5 participants in each group. You can calculate the individual final earnings in a period according to the formula below (we also illustrate the formula with two examples).

**The case when 0 < t ≤ 1**

If t is positive, a fraction equal to t is collected from the money generated by each member in the production task. This means that the group member keeps (1–t) of the money that group member generated in the production task. That is, when t is positive, each group member keeps the sum (1–t)*x_i_, while contributing t*x_i_ to the common pool. This is illustrated in the first term of the payoff equation.

For example, if t = 0.5, half of the money generated (50%) from production is collected from each group member. Each group member keeps the remaining half. Thus, participants who generated more money pay higher amounts to the common pool than participants who generated lower amounts.

The money collected from all five group members is added up, divided into 5 equal parts, and returned to the group members, such that the total sum collected from the 5 group members always equals the sum paid out. This is illustrated in the second term of the payoff equation, t*1/5 ∑x_i_.

Thus, all participants in a group pay different amounts in, which are proportional to their earnings from the task, but get the same amount back. Therefore, if t is positive, group members who generated less money than the average receive more money back than they contribute, whereas those who generated more money than the group average receive less money back than they contribute.

**Example**:

We will now go through an example. Please note that the numbers used in this example are also shown on the table on the next page.

- Assume that the five votes for the transfer parameter are: -0.5, -0.3, 0.4, 0.5 and 0.7.
- The median vote is thus 0.4, and implies that each group member will contribute a share of 0.4 (40 percent) of the money they generated to the common pool.
- Assume further that the ECU generated from production by the five group members are 50, 100, 150, 200 and 250.
- In this case, a value of t = 0.4 means that participant A, who generated 50 ECU in the production task, contributes 20 ECU (0.4*50 = 20) to the common pool, and keeps 30 ECU (50*(1-0.40) = 30). The other group members contribute 40, 60, 80 and 100 ECU, respectively.
- The common pool is then, in total, 300 ECU (20 + 40 + 60 + 80 + 100 = 300).
- The common pool is divided in 5 equal shares of 60 ECU. Each of the five group members receives an equal share.
- Thus, the final earnings for the period for participant A are 50 - 20 + 60 = 90 ECU, for participant B the final earnings are 100 - 40 + 60 = 120 ECU, and so on.

The table below shows you, for each group member, the **ECU generated** in the production task, the **amount collected** from that group member and placed into the common pool, the **amount paid back** to that group member, and the **final earnings** of the group member in the example above.

Since t is greater than zero, group members who generate more money in the production task contribute more to the common pool, and members who generate less money in the production task contribute less, while all members get an equal share back from the common pool.

As you can see, when t is positive, it decreases the payment differences arising in the task.

|  | **A** | **B** | **C** | **D** | **E** |
| --- | --- | --- | --- | --- | --- |
| **Vote for t** | **-0.5** | **-0.3** | **0.4** | **0.5** | **0.7** |
| **Value of t for group (median vote)** |  |  | **0.4** |  |  |
| **ECU generated by production (xi)** | **50** | **100** | **150** | **200** | **250** |
| **Amount**  **collected (Proportional: t*xi)** | **20** | **40** | **60** | **80** | **100** |
|  | *0.4*50=20* | *0.4*100=40* | *0.4*150=60* | *0.4*200=80* | *0.4*250=100* |
| **Total amount collected** | **300** | | | | |
| **Amount paid back (Equal: total amount collected/5)** | **60** | **60** | **60** | **60** | **60** |
|  | *300/5=60* | *300/5=60* | *300/5=60* | *300/5=60* | *300/5=60* |
| **Total amount paid back** | **300** | | | | |
| **Net gain/loss** | **40** | **20** | **0** | **-20** | **-40** |
|  | *60-20=40* | *60-40=12* | *60-60=0* | *60-80= - 20* | *60-100= - 40* |
| **Final earnings** | **90** | **120** | **150** | **180** | **210** |
|  | *50+40=90* | *100+20=120* | *150+0=150* | *200-20=180* | *250-40=210* |

**The case when -1 ≤ t < 0**

If t is negative then 1–t is larger than 1, which means that the first term of the payoff equation, (1–t)*x_i_ is larger than x_i_. This means that the group members receive an additional payment equal in a to t*x_i_. Thus, contrary to the case where t is positive, when t is negative the sum paid out to the group members is proportional to the sum of money they generated in the production task. Group members who produced more money receive a larger payment, and group members who produced less receive a smaller payment.

For example, if t = - 0.5, each of the group members get an additional payment corresponding to 50 percent of the money that group member generated in the production task, or 0.5*x_i_. Thus, participants who generated more money receive higher amounts.

The total money that is to be paid out to the group members is collected in equal shares from all the group members. Specifically, the 5 amounts to be paid out are added up and divided into 5 equal parts. Each group member pays one of these equal shares. This is illustrated in the second term of the payoff equation, t*1/5 ∑x_i_. The amount is equal to the average of the payments made to the five group members.

Thus, all participants in a group get different amounts back, which are proportional to their earnings from the task, but pay the same amount in. Therefore, if t is negative, group members who generated less money than the average receive less money back than they contribute, whereas those who generated more money than the group average receive more money back than they contribute.

**Example**:

We will now go through an example. Please note that the numbers used in this example are also shown on the table on the next page. **[Addition Risk Condition:** In this example, and all subsequent examples, we will assume that the productivity values for everyone in the group are equal to 10. This is to keep the examples simple and keep all numbers as multiples of 10. Note, however, that this will likely not be the case, since productivity values will be randomly determined for all group members in every period, and can vary from 0 to 20.]

- Assume that the five votes for the transfer parameter are: -0.7, -0.5, -0.4, 0.3 and 0.5.
- The median vote is thus -0.4, and implies that each group member will receive an additional payment equal to 0.4 (40 percent) of the money they generated.
- Assume further that the ECU generated from production by the five group members are, as before, 50, 100, 150, 200, 250.
- In this case, a value of t = – 0.4 means that participant A, who generated 50 ECU in the production task, receives 20 ECU (0.4*50 = 20). The other group members receive 40, 60, 80 and 100 ECU, respectively.
- The total sum of payments to the group members is then 300 ECU (20 + 40 + 60 + 80 + 100 = 300).
- The total sum of payments is collected from the group members as 5 equal shares of 60 ECU each (300/5 = 60).
- Thus, the final earnings for the period for participant A are 50 + 20 – 60 = 10 ECU, for participant B the final earnings are 100 + 40 - 60 = 80 ECU, and so on.

The table below shows you, for each group member, the **ECU generated** in the production task, the **additional payment** to that group member, the **amount collected** from that group member, and the **final earnings** of the group member in the example above.

Since t is less than zero, group members who generate more money in the production task receive a larger additional payment, and members who generate less money in the production task receive less, while all members contribute an equal share to the common pool.

As you can see, when t is negative, it rewards those group members who generate more money in the production task.

|  | **A** | | **B** | | **C** | | **D** | | **E** |
| --- | --- | --- | --- | --- | --- | --- | --- | --- | --- |
| **Vote**  **(t)** | **-0.7** | | **-0.5** | | **-0.4** | | **0.3** | | **0.5** |
| **Value of t for group (median vote)** | **-0.4** | | | | | | | | |
| **ECU generated by production (xi)** | **50** | | **100** | | **150** | | **200** | | **250** |
| **Additional payment received (Proportional: t*xi)** | **20** | | **40** | | **60** | | **80** | | **100** |
|  | *0.4*50=20* | | *0.4*100=40* | | *0.4*150=60* | | *0.4*200=80* | | *0.4*250=100* |
| **Total amount paid back** | **300** | | | | | | | | |
| **Amount collected** | **60** | **60** | | **60** | | **60** | | **60** | |
| **(Equal: total amount paid back/5)** | *300/5=60* | *300/5=60* | | *300/5=60* | | *300/5=60* | | *300/5=60* | |
| **Total amount collected** | **300** | | | | | | | | |
| **Net gain/loss** | **-40** | **-20** | | **0** | | **20** | | **40** | |
|  | *20-60= - 40* | *40-60= - 20* | | *60-60=0* | | *80-60=20* | | *100-60=40* | |
| **Final earnings** | **10** | **80** | | **150** | | **220** | | **290** | |
|  | *50-40=10* | *100-20=80* | | *150+0=150* | | *200+20=220* | | *250+40=290* | |

**Three special cases**

Below we further illustrate the effects of t by three special cases:

- If t = 1, 100 percent of the ECUs the group members generate through production is collected and transferred back in equal amounts. Thus, if t = 1, all group members end up with the same final earnings in a period, regardless of how much money they generate from the production task.

In the example above, the group members generated 750 ECU from the production task in total. If t = 1, the entire 750 would be collected into the common pool and each group member would thus receive a final payoff of 750/5 = 150 ECU.

- If t = 0, nothing is collected from the money the group members generate from production. In this case, each group member receives, as final payoff, the amount that group member generated from production in the task.
- If t = -1, the final earnings for the group members who generated the least money in the production task may end up being negative. However, earnings may not be negative for any participant or period. Therefore, we restrict t such that the lowest earnings possible in a period will be 0. If the median vote for t ends up, at the end of the period, leaving any group member with negative final earnings in that period, then the computer adjusts t upwards until no group member is left with negative earnings.

  For example, assume that in the example above t = -1. This implies that each group member receives an additional payment equal to the amount that group member generated from production. The group member who generated 50 ECU receives an additional payment of 50 ECU. This also means that a total of 750 ECU is paid out and an equal sum of 750/5 = 150 ECU is collected from each group member. The final earnings of the group member who generated 50 ECU would then be 50 + 50 – 150 = -50 ECU. Hence with this distribution of money generated from production, a value of t equal to -1 cannot be implemented, since earnings cannot be negative. Instead, the lowest value of t that leaves no group member with negative earnings will be implemented. In this example, the lowest value of t that satisfies this requirement is t = -0.5, which leaves the least productive group member with final earnings of 0 ECU.

Before we continue, you will have the opportunity to familiarize yourself with how t influences final payoffs. Please click “Continue” on your screen now. You will then see an example of a practice screen in which you can test the effect of different values of t on the distributions of final earnings.

You now see an example of the practice screen. At present, you cannot insert any values. Once I describe the screen, you will again be asked to click “Continue” and the real practice screen will appear.

The practice screen allows you to enter various values of t, and see how these values influence the redistribution and final earnings, for different amounts of money generated by the members of a group.

In the upper part of the screen, under “Transfer parameter” you can fill in hypothetical values for the transfer parameter, t. During the experiment, the transfer parameter will be decided by the median vote cast by the group’s members.

In the table below, under “Redistribution” you can fill in hypothetical amounts of money generated in the production task for each of the five group members.

Once you have filled in hypothetical values, you can click “OK”, and the table then displays, for each group member, the amount collected from that group member, the amount of money received by that group member, the corresponding net transfer, and the final earnings for that group member.

Remember that a positive value of t benefits group members who generated less money than the group average, and a negative value of t rewards group members who generated more money in the task than the group average.

Please now press “Continue” to get to the practice screen. You will then have 3 minutes to test different combinations of t and the money generated by group members. If you need a calculator, you can click on the symbol in the lower right corner, and a calculator will become visible.

**Information about current and previous periods**

In each period, after all group members vote on the value of t in a period, you will see the transfer parameter, t, for your group for that period. You will see this before starting the production task for that period.

In addition, at the end of each period you will see a feedback screen with information about the outcome of the current period for all members of your group. [**Addition Risk Condition**: At this point, you will also be told of your productivity value for that period.]

**[No Risk Condition]** Specifically, you will see the value of the transfer parameter, as well as a table including the amount of money each group member generated from production, the group member’s rank in the group for that period, and the net transfer and final earnings for each group member. For each group member, you will also see the average amount of money produced by that group member across all previous periods.

**[Risk Condition**] Specifically, you will see the value of the transfer parameter, as well as a table including the number of sequences each group member completed, each group member’s productivity value for that period, the amount of money each group member generated from production, the group member’s rank in the group for that period, and the net transfer and final earnings for each group member. For each group member, you will also see the average amount of money produced by that group member across all previous periods

The information screen will also provide the transfer parameter and all group members’ production and final earnings for all periods. This will be at the bottom of the screen, in a scrollable box.

In addition, at the beginning of each period, before you vote for a value of t, a screen will be available for up to 60 seconds where you may calculate the effect on final earnings due to different values of t. On this screen, you can also find the results of previous periods, as well as individual average production and earnings across previous periods.

**Comprehension Questions**

Before starting the first period of Part 3, we would like you to answer a few questions to ensure everyone understands the instructions. Please click the “Continue” button on your screen now. You will then see a table with an example of the votes for a hypothetical group. A few cells in this table are empty.

First, we ask you to fill in the transfer parameter, given the hypothetical votes given by the group members. Please remember that the median is the value that lies in the middle when the five votes are ordered from highest to lowest.

Once you have confirmed the correct answer, the next part of the table, which displays the outcomes of the production task, appears. Please continue entering the correct amounts collected or paid back to the group members where this information is missing. In total, you will see two different examples.

If anything is unclear, please raise your hand and an experimenter will assist you. When everybody has answered the comprehension questions Part 3 will start.
